# Parity associates with chromosomal damage in uterine leiomyomas

**DOI:** 10.1038/s41467-021-25806-x

**Published:** 2021-09-14

**Authors:** Heli Kuisma, Simona Bramante, Kristiina Rajamäki, Lauri J. Sipilä, Eevi Kaasinen, Jaana Kaukomaa, Kimmo Palin, Netta Mäkinen, Jari Sjöberg, Nanna Sarvilinna, Jussi Taipale, Liisa Kauppi, Manuela Tumiati, Antti Hassinen, Janne Pitkäniemi, Jyrki Jalkanen, Sanna Heikkinen, Annukka Pasanen, Oskari Heikinheimo, Ralf Bützow, Niko Välimäki, Lauri A. Aaltonen

**Affiliations:** 1 Department of Medical and Clinical Genetics and Applied Tumor Genomics Research Program University of Helsinki, Helsinki, Finland; 2 Department of Obstetrics and Gynecology, University of Helsinki and Helsinki University Hospital, Helsinki, Finland; 3 Systems Oncology Research Program, University of Helsinki, Helsinki, Finland; 4 FIMM-HCA, Institute for Molecular Medicine Finland (FIMM), Helsinki, Finland; 5 Institute for Statistical and Epidemiological Cancer Research, Finnish Cancer Registry, Helsinki, Finland; 6 Faculty of Social Sciences, University of Tampere, Tampere, Finland; 7 Department of Public Health, University of Helsinki, Helsinki, Finland; 8 Department of Obstetrics and Gynecology, Central Finland Central Hospital, Jyväskylä, Finland; 9 Department of Pathology, University of Helsinki and Helsinki University Hospital, Helsinki, Finland

**Keywords:** Cancer genetics, Cancer genomics

## Abstract

Mechanical forces in a constrained cellular environment were recently established as a facilitator of chromosomal damage. Whether this could contribute to tumorigenesis is not known. Uterine leiomyomas are common neoplasms that display relatively few chromosomal aberrations. We hypothesized that if mechanical forces contribute to chromosomal damage, signs of this could be seen in uterine leiomyomas from parous women. We examined the karyotypes of 1946 tumors, and found a striking overrepresentation of chromosomal damage associated with parity. We then subjected myometrial cells to physiological forces similar to those encountered during pregnancy, and found this to cause DNA breaks and a DNA repair response. While mechanical forces acting in constrained cellular environments may thus contribute to neoplastic degeneration, and genesis of uterine leiomyoma, further studies are needed to prove possible causality of the observed association. No evidence for progression to malignancy was found.

## Abstract (web summary)

Many factors have been associated with chromosomal damage, including mechanical forces in a constrained cellular environment. Here the authors reveal an association between parity and chromosomal damage by analysing karyotypes of 1946 uterine leiomyomas.

## Introduction

The notion that confined environment is a cause of chromosomal damage has attracted significant attention in recent years and rupture of the nuclear envelope (NE) as a mechanism has been in a particular focus^1,2^. NE rupture has been found to be a common phenomenon even under normal conditions, and the frequency increases dramatically in confined environments^3^. Known causes for NE rupture include increased actomyosin contractility often promoted by the stiffness of the extracellular matrix^4,5^, as well as migration through narrow pores (reviewed in^6^). Damage can also be caused by nuclear deformation without NE rupture, for instance by mechanical shearing of DNA or exclusion of DNA repair factors from the site of DNA damage^7,8^. In addition, nuclear deformation caused by migration and cell compression have been shown to cause DNA damage via increased replication stress without NE rupture^9^. While chromosomal damage is an important driver of tumorigenesis^10^, whether mechanical forces could be a significant contributor to human neoplasia has not yet been addressed.

During pregnancy uterine smooth muscle cells are subject to constant stretching and subsequently, during labor, forceful episodic contractions generated by actin and myosin^11,12^. Uterine leiomyomas (ULs) are extremely common benign smooth muscle tumors. Lifetime prevalence approaches 70%^13^. ULs are a frequent cause of abnormal bleeding and pain, and are the most common cause of hysterectomy. Occurrence of multiple synchronous tumors is typical^14^. Most ULs are driven by a mutation in the *MED12* gene^15,16^. The karyotype is typically relatively stable as compared to the often highly complex karyotypes seen in cancer, yet chromosomal aberrations due to DNA double-strand breaks (DSBs) – in particular losses of chromosomal regions – are commonly seen^17–19^. A particularly interesting feature is the relatively frequent occurrence of complex chromosomal rearrangements (CCRs) that resemble chromothripsis, in otherwise benign ULs^17^. Chromothripsis is a single event where one or few chromosomes become subject to tens to hundreds of rearrangements. It is usually associated with *TP53* mutant cancers^20^. Multiple mechanisms may lead to this phenomenon, the formation of micronuclei and subsequent damage to the chromosomes entrapped in them being a likely key cause^21^. The CCRs observed in ULs involve a limited number of chromosomes, encompass chained structural rearrangements, and thus, appear to have formed in a single event as shown in^17^. While these features characterize also chromothripsis, the difference is that most of the CCR events detected in UL are less dramatic. One plausible explanation is that the chromothriptic events that are powerful cancer drivers are not tolerated in UL genesis, as the cellular checkpoints are still intact^21^ in the *TP53* wild-type precursor cells^17^. Uterine leiomyosarcoma (ULMS) is a rare malignant smooth muscle tumor displaying prevalent copy-number changes and chromothripsis events^22^. While the malignant potential of UL is extremely low, the possibility of transformation of UL to ULMS has been proposed^23^.

In this work, we test the hypothesis that if mechanical forces acting in a constrained cellular environment are a relevant source of chromosomal damage, signs of this could be seen in myometrial tumors from women who have undergone pregnancy and delivery. We examine the association of parity to chromosomal breakage in 1946 ULs derived from 628 patients, as well as to the association to ULMS in a population-based setting.

## Results

### Patient and tumor characteristics

**Fig. 1:**
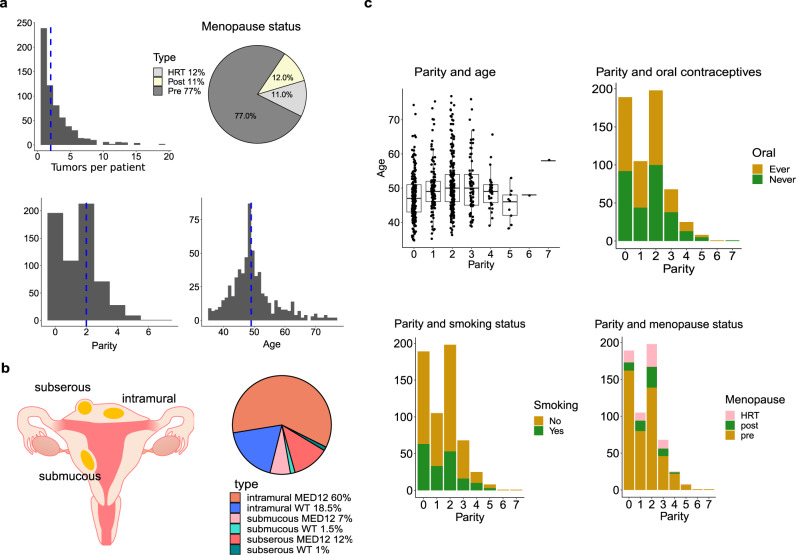
Patient characteristics and tumor location. **a** Patient characteristics (*N* = 628). The distribution of the number of tumors per patient, parity, the age of the patients, and menopause status. Median is shown by blue vertical line. **b** Intramural, subserous, and submucous locations of uterine leiomyomas (ULs) (*N* = 1451). The majority of the tumors are intramural, but *MED12* mutated tumors are associated with subserous location. **c** On the top left, age at hysterectomy stratified by parity. The *y*-axis is in years. Boxplots show the median and the first and third quartiles. Error bars extend up to 1.5 IQR above the quartiles. The number of patients in each class is: parity 0 = 196, 1 = 108, 2 = 213, 3 = 71, 4 = 28, 5 = 9, 6 = 1, 7 = 1. On the bottom left, the number of patients with a history of ever smoking (yes/no) stratified by parity. On the top right, the number of patients with combined oral contraceptives use (ever/never) stratified by parity. On the bottom right, the number of pre-menopausal, post-menopausal patients and patients with hormonal replacement therapy (HRT). Source data are provided.

Parity was known for 628 patients, and 1935 tumors entered the analyses. In all, 31% of the tumors were from nulliparous patients and 69% from parous patients; 79% of the tumors were *MED12* mutated (referred as MED12 tumors); and 21% were wild type (WT) (referred as WT tumors) for known *MED12* driver mutations. A total of 595 patients contributing 1836 tumors had information on all background variables: age at hysterectomy, menopause status (pre, post, and hormonal replacement therapy), hormonal contraception use (never/ever), and smoking status (ever/never). Summary of patient characteristics is reported in Fig. 1a and Supplementary Table 1, tumor characteristics in Fig. 1b, and relationship between parity and the background variables in Fig. 1c.

### Chromosomal gains and losses in UL associate with parity

**Fig. 2:**
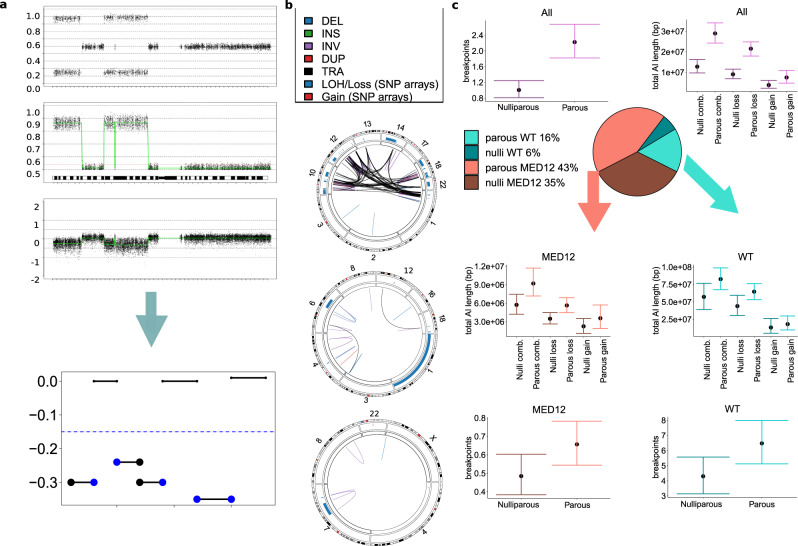
Overview tumor characteristics. **a** Allelic imbalance (AI) segments in SNP-array data. From top-down, examples of chromosome 1 B-allele frequencies (BAF), mirrored BAF (mBAF) and Log R Ratios (LRR). Bottom, a schematic figure of the interpretation of the segments: black horizontal lines represent AI segments. Blue horizontal line represents LRR threshold −0.15. Blue dots represent the calculated breakpoints: I. When AI segment is at the end of the chromosome, only one breakpoint is counted for. II. If the LRR difference between adjacent copy-number segments is <0.1, the segments are considered contiguous and are counted as only two breakpoints. III. When the segment is not at the chromosome end and the LRR difference between both adjacent segments is >0.1, two breakpoints are calculated. **b** Typical allelic imbalance patterns in ULs. Representative examples of complex chromosomal rearrangement (CCR) patterns in different ULs. Only chromosomes affected by allelic imbalance changes are shown: Top: the majority of tumors with high breakpoint count are from tumors wild type (WT) for known *MED12* mutations. Translocations between chr12 (*HMGA2*) and chr14 (*RAD51B*) are common. Middle: chromosomal rearrangements seen in MED12 tumors are typically more moderate. Bottom: whole-chromosome losses were predominantly detected in parous patients’ WT tumors. Blue line: deletion, green line: insertion, purple line: inversion, red line: duplication, black line: translocation, blue box: LOH/Loss, red box: Gain. **c** Average breakpoint number, total length of loss regions, total length of gain regions, and the combined length of loss and gain regions are presented for tumors from parous and nulliparous patients. The results are presented for all 1935 tumors (purple) and separately for *MED12* (salmon) and WT (turquoise) tumors. 95% bootstrap confidence intervals are presented for the mean. In all comparisons, the mean length of tumor genome AI regions is higher in parous than nulliparous patients. Source data are provided.

We studied the relationship between parity and DNA damage by utilizing single-nucleotide polymorphism (SNP)-array based allelic imbalance (AI) data created in the Finland myoma study^19^. An example of AI determination from SNP-array is presented in Fig. 2a and examples of different levels of DNA damage in WGS data Fig. 2b. The mean number of breakpoints, and the mean of total length of loss regions, total length of gain regions, and total combined length are reported in Fig. 2c and Supplementary Table 2. The results are presented by parity (nulliparous and parous) as well as by tumor subtype (*MED12* mutant and WT). We found an increase in the mean of breakpoint number as well as total length of copy number alterations when tumors from parous patients were compared to tumors from nulliparous patients. For example, the total length of somatic losses was higher in tumors from parous patients than in tumors from nulliparous patients: the mean was 62% higher in MED12 tumors and 47% higher in WT tumors.

The statistical associations are presented in Table 1 and the full models in Supplementary Figs. 1–3. Briefly, the combined total length of loss and gain regions was significantly associated with parity (*P* < 0.001, rate ratio (RR) = 1.32, 95% CI: 1.15–1.52, Bonferroni adjusted). The association remained significant within the MED12 tumors (*P* = 0.007, RR = 1.39 95% CI: 1.05–1.84).

**Table 1:** Association between chromosomal damage and parity.

Response^a^	Tumors^b^	*n* tumors	*n* patients	*P* (adjusted^c^)	Model	Effect [95% CI]^d^ (adjusted^c^)
Total AI region length (bp)	All	1836	595	1.02e-07	Poisson	1.32 [1.15–1.52]
Total AI region length (bp)	*MED12*	1442	429	0.0088	Poisson	1.39 [1.05–1.84]
Total AI region length (bp)	WT	394	306	1	Poisson	1.05 [0.9–1.22]
CCR status (yes/no)	All	1836	595	0.000154	Binomial	1.29 [1.09–1.52]
CCR status (yes/no)	*MED12*	1442	429	0.00572	Binomial	1.465 [1.07–2.0]
CCR status (yes/no)	WT	394	306	1	Binomial	0.953 [0.754–1.2]
Whole chromosome loss (yes/no)	WT	394	306	0.0015	Binomial	1.64 [1.13–2.36]

*bp* base pairs, *AI* allelic imbalance, *CCR* complex chromosomal rearrangements.

^a^Parity is the predictor. Confounders: history of ever smoking (yes/no), age, menopause status (pre/hormonal replacement therapy/post), and combined oral contraceptive use (never/ever).

^b^All: all tumors regardless of mutation status; *MED12*: only the MED12 mutation-positive tumors; WT: only the MED12 mutation-negative tumors.

^c^Bonferroni adjusted two-sided P-values and 95% confidence intervals (adjusted alpha 0.05/11).

^d^For binomial regression, odds ratio is reported and for Poisson regression rate ratio.

**Fig. 3:**
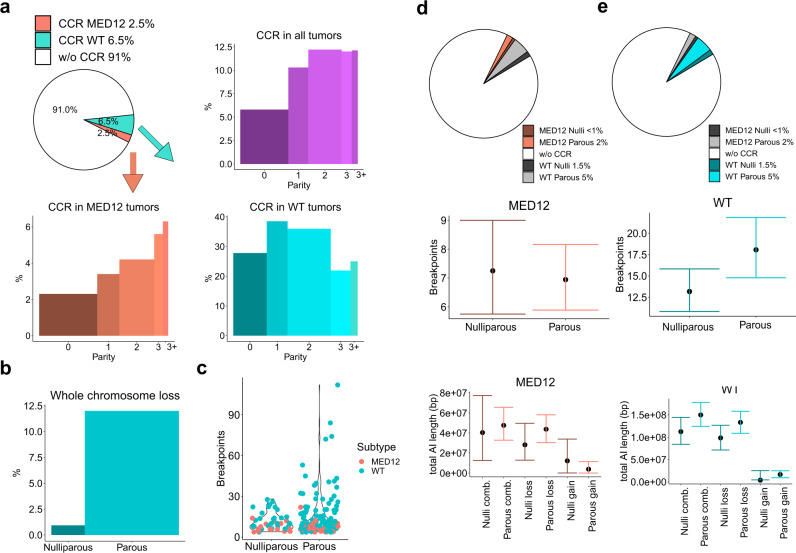
Overview of complex chromosomal rearrangements (CCRs). **a** Summary of CCR statuses for all 1935 tumors (purple), stratified by patient’s parity. Similarly, CCR statuses among *MED12* mutated tumors (salmon) and among WT tumors (turquoise). **b** Association between whole chromosome loss and parity. **c**, Breakpoint number in tumors with CCR, stratified by nulliparous (*n* = 45 tumors) and parous patients (*n* = 134 tumors). Orange dots depict *MED12* mutated tumors, turquoise WT tumors. Of note, all ULs with a high breakpoint number are from parous patients’ WT tumors. The mean number of breakpoints in CCR–nulliparous tumors is 13.0 (95% CI: 10.93–15.79) and in CCR–parous tumors 18.17 (14.74–22.14). **d**, An average breakpoint number, total length of loss regions, total length of gain regions and the combined length of loss and gain regions are presented for *MED12* tumors with CCR (*n* = 52) from parous (*n* = 36) and nulliparous patients (*n* = 16). 95% bootstrap confidence intervals are presented for the mean. **e** An average breakpoint number, total length of loss regions, total length of gain regions and the combined length of loss and gain regions are presented for WT CCR tumors (n = 127) from parous (*n* = 98) and nulliparous patients (*n* = 29). 95% bootstrap confidence intervals are presented for the mean. Source data are provided.

Forty tumors had at least one whole chromosome loss. Of these tumors, only two (5%) were *MED12* mutated. The most common loss, observed in 19 tumors, was chr22, followed by chr13 and chr14 in seven tumors. The WT tumors displayed a significant positive association between whole chromosome losses and parity (*P* = 0.002, odds ratio (OR) = 1.64, 95% CI: 1.13–2.36). See Supplementary Fig. 4 for full model. Among nulliparous patients, only one tumor displayed whole chromosome losses (Fig. 3b).

### Identifying complex chromosomal rearrangements

CCR events in 38 whole-genome sequenced (WGS) ULs were readily available^17^. We studied the number of DSBs in WGS samples with and without CCR as well as the respective SNP-array data, and extrapolated the CCR criteria to all SNP-array samples. The SNP-array based analysis identified 179 tumors (9.3% out of 1935) compatible with CCR. The mean number of breakpoints, and the total length of copy-number losses and gains are reported in Supplementary Table 2 for both CCR tumors and tumors without CCR separated by parity and subtype. *MED12* mutation-positive tumors were underrepresented among the CCR tumors (29.0% out of 179), in line with our previous study^17^.

SNP-array based predicted CCRs were validated in 43 tumors by long-read WGS. Three tumors (7%) did not display interconnected rearrangements, while 40 (93%) displayed at least one CCR event (Supplementary Fig. 5 and Supplementary Table 3). Three tumors with CCR and different patterns of chromosomal damage are presented in Fig. 2b.

### CCRs in UL associate with parity

Parity was significantly associated with CCR events (Fig. 3a, *P* < 0.001, OR = 1.29, 95% CI: 1.09–1.52). The association remained significant within the MED12 tumors (Fig. 3a, *P* = 0.006, OR = 1.47, 95% CI: 1.07–2.0). The confounding variables—age, smoking status, menopausal status and use of oral hormonal contraceptives—were not associated with CCR. See Supplementary Figs. 6–8 for full model description. Among the 179 tumors affected by CCR, the number of breakpoints was larger in tumors from parous patients compared to tumors from nulliparous patients: all 16 tumors with more than 28 breakpoints were from parous patients (Fig. 3c). The total length of AI for CCR tumors is presented in Fig. 3d, e.

### The effect of tumor location on allelic imbalance

Information on tumor location and size was available for 1451 tumors. From the 1142 MED12 tumors 76% were intramural, 15% subserous, and 9% submucous. From the 309 WT tumors 87% were intramural, 6% were subserous, and 7% were submucous (Fig. 1b). In MED12 tumors, shorter length of lost chromosomal regions was observed in subserous tumors compared to intramural tumors (*P* = 0.001, RR = 0.28, 95% CI 0.11–0.72, Supplementary Fig. 9 and Supplementary Table 4). Tumor size was not associated with AI length.

### Mechanical force induces DNA damage in myometrial cells

**Fig. 4:**
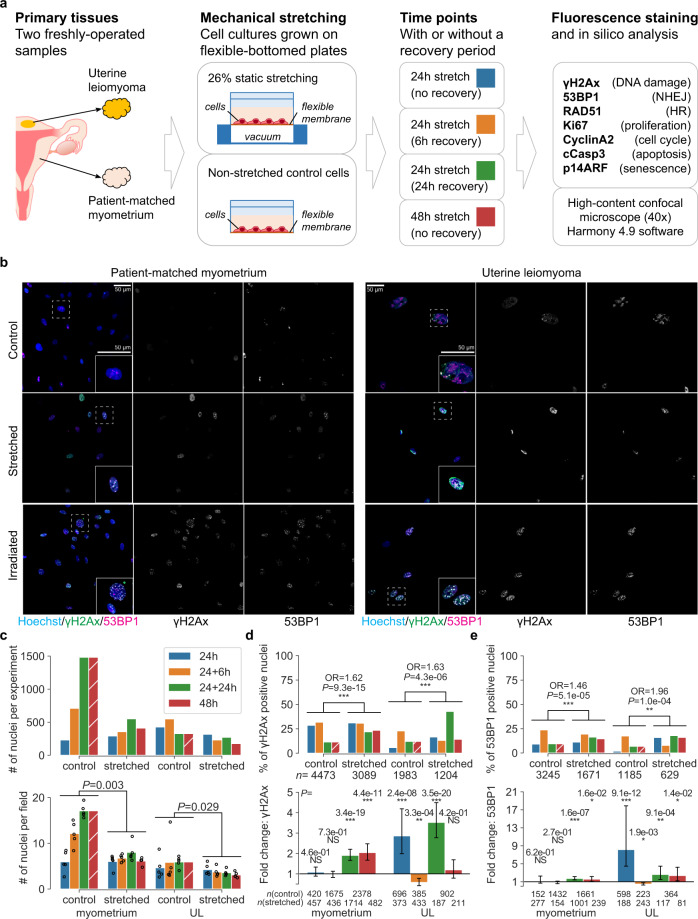
Exposing uterine leiomyoma and myometrium cells to mechanical stretch induces DNA damage and non-homologous end joining (NHEJ)-mediated repair. **a** Schematic overview of the experiment. HR homologous recombination. **b** Representative fields of view of cells under each experiment condition (stretched and non-stretched control at the 24 h stretch+24 h recovery time point). Inset shows individual nuclei at higher magnification. **c** Number of nuclei quantified in the in silico image analysis. On top, average numbers of nuclei per experiment condition. On bottom, average numbers of nuclei per one imaging field (bar plot is mean of means; dots are mean values per experiment). Unadjusted, two-sided Mann–Whitney *U* test *P*-values shown between control (*n* = 15) and stretched (*n* = 20). Blue: 24 h stretch without recovery; orange: 24 h with 6 h recovery; green: 24 h with 24 h recovery; red: 48 h without recovery. Time points 24 + 24 h and 48 h share the same control (hatched red bar). **d** DNA damage (γH2Ax positive nuclei) and, **e** NHEJ repair (γH2Ax + 53BP1 positive nuclei) quantified in stretched and non-stretched control cells. **d**, **e** Data are presented as a proportion (%) of positive nuclei (top bar plots) and as a fold-change of nuclei-positivity (bottom bar plots) between stretched and non-stretched control cells; error bars show a bootstrapping 95% confidence interval of each fold-change value. Odds ratio (OR) and fold-change (FC) values >1 suggest that stretching induces DNA damage/repair. Unadjusted, two-sided *P*-values from either logistic regression (top) or Fisher’s exact test (bottom) are denoted by: ***P < 0.0001, **P < 0.001, *P < 0.05, not significant (NS) *P* ≥ 0.05. Sample sizes (*n*) are shown for each significance test. See Supplementary Tables 6–7 for multiple-test adjusted *P*-values. Source data are provided as a Source Data file.

Effects of mechanical stretching were studied using primary cell cultures from patient-derived uterine leiomyoma and corresponding normal myometrium. Static stretching with 26% elongation was applied to the cells cultured on flexible membranes for 24–48 h with or without a recovery period (Fig. 4a). After releasing the stretch, initial evaluation by light microscopy showed the typical elongated morphology in non-stretched control cells, while the stretched cells appeared smaller and rounder. After fixation and immunofluorescence stainings, the cells were imaged by high-content confocal microscopy for in silico analysis. Representative fields of view of the cells are shown in Fig. 4b. An average of 483 nuclei were identified in the image analysis per staining (median 282 nuclei; interquartile range of 183–571 nuclei quantified per staining; Fig. 4c; details in Supplementary Fig. 10 and Supplementary Table 5).

To evaluate the level of DNA damage, γH2Ax positive nuclei (>10 γH2Ax foci) indicating DNA DSBs were quantified in stretched cells compared to non-stretched control cells. Myometrium and leiomyoma cells treated with ionizing irradiation (IR) as a positive control displayed the expected increase in γH2Ax positive nuclei (OR = 33.1 and OR = 11.9, respectively; both *P* < 0.0001; Supplementary Fig. 11). Stretching resulted in a more moderate yet highly significant increase in γH2Ax-positive nuclei both in leiomyoma and myometrium cells (OR = 1.6 and *P* < 0.0001; Fig. 4d and Supplementary Fig. 12). The response was dependent on treatment time; significant DNA damage was first detectable after 24 h of stretching in leiomyoma cells and showed a biphasic pattern, while myometrium cells showed significant DNA damage at the two later time points (Supplementary Table 7). The increase in DNA damage coincided with an increase in DNA repair (Fig. 4e). Non-homologous end joining (NHEJ), quantified as nuclei double-positive for γH2Ax (>10 foci) and 53BP1 (>5 foci), was the major DNA repair mechanism induced by stretching in both myometrium (OR = 1.5) and leiomyoma (OR = 2.0; both *P* < 0.0001; Fig. 4e and Supplementary Fig. 13) cells. Nuclei double-positive for RAD51 (>5 foci) and cyclinA2 (cell cycle S/G_2_), indicating homologous recombination (HR), were rare in both the IR (Supplementary Fig. 11) and stretching experiments (Supplementary Fig. 14, Supplementary Fig. 15), with no statistically significant differences between the experimental conditions. Taken together, mechanical stretching induced both DNA damage and NHEJ repair (Supplementary Table 6).

Reduced cell density was observed after stretching in both myometrium and UL cell cultures (*P* < 0.05; Fig. 4c). This was not due to apoptosis (as measured by cleaved caspase-3 positivity) that predominantly showed a decrease, perhaps owing to additional space created by stretching (Supplementary Fig. 16). While stretching had no overall effect on cell senescence (p14ARF) (Supplementary Fig. 17), it reduced the number of proliferative cells (nuclear Ki67 foci) (Supplementary Figs. 15 and 18). There was no consistent effect on numbers of cyclin A2 positive nuclei, that is, S phase cells (Supplementary Figs. 15 and 19). Notably, even after 48 h of stretching, a considerable proportion (25–37%) of the surviving cells remained proliferative. Data on CyclinA2, Ki67, cCasp3 and p14ARF are summarized in Supplementary Table 8. Segmented nuclei imaging data revealed increased roundness in nuclei with >10 versus <10 γH2Ax foci after stretching (*P* < 0.0001; Supplementary Fig. 20); also nuclear area showed a slight increase in γH2Ax positive stretched cells.

### Parity is not associated with uterine leiomyosarcoma risk

The population-based analysis of 399 ULMS patients and 1657 matched controls did not show a statistically significant association (OR = 1.03; 95% CI 0.96–1.11; *P* = 0.38) between parity and ULMS.

## Discussion

Confined cellular environment is a recently proposed source of chromosomal damage^2,7,24^. It has also been shown that many cells are viable after mechanical stress^7^ and thus, the damage-induced chromosomal aberrations can be passed onto daughter cells. We hypothesized that traces of this phenomenon might be detected in uterine leiomyomas from parous women, as myometrial cells are exposed to mechanical forces during pregnancy and parturition and chromosomal aberrations have the potential to drive neoplastic growth. Our data shows that chromosomal breakage in UL is strongly associated with parity. We studied both the total length of genome altered by copy-number changes, a commonly used surrogate for genomic instability^25,26^, and the occurrence of complex chromosomal rearrangements resembling chromothripsis. Both of these DNA damage types displayed a positive association. Considering that the mechanisms that cause structural and numerical chromosome instability may be different^27^, we analyzed the occurrence of whole chromosome losses and found also these to be significantly associated with parity. Because chromosomal instability is an established feature of many cancers including ULMS, we examined the association of parity to occurrence of this malignancy, with negative results. While our population-based ULMS patient series is extensive considering the rarity of the condition^22,23^, its power was limited to large effects (OR ≥ 1.5), and even more extensive material is required to comprehensively resolve the question. The possible increased risk of ULMS associated with parity is likely to be small, if at all present.

We examined experimentally, utilizing static stretching of primary myometrial and leiomyoma cells, whether forces similar to those observed during pregnancy have the potential to cause DNA damage. The stretched cells displayed a significant increase in DNA damage, and concomitant induction of DNA repair by NHEJ was detected. This result is compatible with previous observations of cyclic mechanical tension triggering an increase in γH2Ax foci in the inner core of the vertebral disc^28^. Moreover, cyclic stretching was previously shown to enhance (though not induce) nuclear rupture and DNA damage caused by lamin A knockdown in cardiomyocytes^4^. In our experiments, stretching resulted in reduced cell density in the absence of apoptosis; one contributing factor may be the impaired cell proliferation observed after stretching. Nevertheless, a proliferating subpopulation of cells persisted after the challenge, and there was no induction of cell senescence in these short-term experiments.

Of the main pathways repairing double-strand breaks^29^, NHEJ was the major repair mechanism induced by stretching, while HR-mediated repair was uncommon both after irradiation and stretching. Because NHEJ does not use a complementary template, the fusion of two broken ends of DNA duplexes, with no or limited homology, may result in deletion or insertion of base pairs and other rearrangements^30,31^. Thus, occasional transmission of genetic alterations to daughter cells after stretching-induced DNA damage seems conceivable, although the short-lived primary cell cultures precluded long-term follow-up. This could be involved in increasing the risk of developing ULs after mechanical stretching. Further studies are needed to clarify what mechanisms contribute to the low rate of HR in human myometrium and ULs.

The irradiated positive control cells cultured on glass showed a more robust induction of γH2Ax foci compared to the stretched cells on flexible membranes. Compatible with these observations, increasing stiffness of cell culture matrix favors DNA damage and nuclear rupture in nuclear lamin A knockdown cells^4,5^. In this model, cell spreading on a stiff matrix causes local sites of high nuclear curvature, promoting rupture and decreasing nuclear circularity in live imaging^5^. In our experimental setup where the cells were fixed after the release of stretching, the stretched cells positive for DNA damage showed an increased roundness. While these observations may be related to nuclear deformation occurring during stretching or recovery, the detailed mechanisms remain to be elucidated in future studies. Interestingly, nuclear deformation, even in the absence of nuclear envelope rupture, can cause DNA damage through replication stress in the context of cell compression or migration through tight spaces^9^. Taken together, cell nuclei are sensitive to several types of mechanical stresses and the responses elicited depend on the cell type, culture matrix, and the type, strength and duration of such stress.

While complete understanding of the association between parity and chromosomal damage in UL needs more work as uterine myometrium is under multiple different powerful cues, direct force acting upon myometrial nuclei during gestation and labor appears as one conceivable candidate factor contributing to our findings in primary tumors. Confined environment is known to predispose cells to DNA damage through multiple different mechanisms (reviewed in Shah et al.^3^): Damage from increased actinomyosin contractility – a known key contributor of nuclear envelope rupture^6^ – that is most prominent at labor, and deformation of nuclei and subsequent damage at nuclear envelope through stretching during gestation, are both plausible possibilities. Mechanical shearing of DNA can take place even without damage to the nuclear envelope^3^. Nuclear deformation and NE rupture are common under extreme conditions, and chromatin protrusion through the nuclear lamina exposes DNA to the cytoplasm, resulting in DNA damage. NE rupture also exposes DNA to cytoplasmic nucleases. A particularly interesting form of damage resembling formation of micronuclei is that after NE rupture chromatin can become fragmented and separated from the nucleus^6^. This could predispose to the CCR changes observed in ULs^17^. The chromosomal fragments following NE rupture may also be distributed unequally during mitosis. Indeed, a defective nuclear envelope has been proposed as a main cause of aneuploidy in ovarian cancer^32^. In addition, mechanical forces work in concert with other cues present in the myometrium; during hypoxia for example^33^.

While our results firmly associate parity to chromosomal damage in UL, further studies are needed to examine whether mechanical forces play a causative role in this association. While mechanical force has recently been established as a factor capable of causing DNA damage^3^, it has to our knowledge not been implicated in genesis of common tumors. Its potential contribution to neoplastic degeneration in myometrium as well as other tissues is an intriguing new avenue of research, towards a more complete understanding of tumorigenesis.

## Methods

### Study subjects

The study was conducted in accordance with the Declaration of Helsinki and approved by the Finnish National Supervisory Authority for Welfare and Health, National Institute for Health and Welfare (THL/151/5.05.00/2017, THL/723/5.05.00/2018), and the Ethics Committee of the Hospital District of Helsinki and Uusimaa (HUS/2509/2016).

Prospectively collected hysterectomy samples were utilized in the study; altogether 2131 uterine leiomyomas (ULs) and 690 corresponding normal myometrium tissue samples (the typical patient thus contributing on average 3 tumors to the study). See below details on the numbers of samples that passed data quality control. Five of the six sample series (M, My, My1000, My5000, and My6000) have been described in more detail in our previous studies^34–36^. An anonymous sample series (‘M’-samples, *n* = 209) was collected according to Finnish laws and regulations after authorization from the director of the health-care unit, between the years 2001 and 2002. For the five subsequent sample series, a written informed consent was obtained from all patients before they entered the study.

In the “My8000” sample series uterine leiomyoma (*n* = 91) and corresponding normal myometrium tissue samples were collected as fresh frozen samples from patients (*n* = 30) with ultrasound-diagnosed uterine leiomyomas and who underwent hysterectomy in the Central Finland Central Hospital between September 2014 and December 2015. All the specimens underwent routine diagnostic pathological scrutiny, and the histopathological diagnoses were retrieved from pathology reports. The smallest lesion collected had a diameter of 4 mm. One patient’s tissue samples were excluded from the study as no myomatous tissue was found in the pathologist’s evaluation. A written informed consent was obtained from all patients before they entered the study.

To examine a possible connection between parity and ULMS, we systematically harvested the Finnish Cancer Registry^37^ database for cases diagnosed with ICD-O-3 topography code 54.2 or 54.9, and morphology code 8890/3. This resulted in a population-based series of 615 ULMS patients for which we retrieved the number of offspring from Population Information System (PIS). With Population Register Centers (PRC)’s approval (VRK/5049/2018-3), we requested child count information for five matched controls per ULMS patient from PRC. Eligible controls had to be women and alive at the time of diagnosis of the ULMS patient. Controls were matched by municipality at birth and birth date, with a time window of 6 months before or after the index ULMS patient’s birth. Of the 615 ULMS patients, 8 were excluded from the analysis due to birth place being abroad or unknown, preventing matching, and 75 were excluded due to lack of any suitable controls. Due to previous linking of nulliparity as a major risk factor in breast, ovarian, and uterine cancers, the analysis set was further restricted to parous patients with at least one parous control, leaving 399 cases and 1657 controls. Mean age at diagnosis for parous patients was 57.8 years, with first and third quartiles at 48 years and 67 years, respectively. Our analysis provided a statistical power of 0.8 for an effect size 1.5.

### Determination of *MED12* driver mutation status

DNA was extracted from all the consecutively collected normal and tumor specimens by standard methods. *MED12* exon 1 and 2 - covering all UL-related mutations thus far reported - were screened for mutations by Sanger sequencing of all 2131 tumors using 5′ to 3′ primers CCTCCGGAACGTTTCATAGAT (forward) and TTCGGGACTTTTGCTCTCAC (reverse), and GCCCTTTCACCTTGTTCCTT (forward) and TGTCCCTATAAGTCTTCCCAACC (reverse), for exon 1 and exon 2, respectively.

### Identifying somatic copy-number aberrations

All tumor and normal pairs were genotyped with Infinium HumanCore-24 (Illumina) SNP-arrays. B-allele frequencies (BAF) and log-R ratios (LRR) were extracted with Illumina GenomeStudio software (version 2011.1; Genotyping module v1.9). After GC wave adjustment of the data with PennCNV (v1.0.4)^38^, allelic imbalance (AI) regions were calculated for all tumors using BAF segmentation (v1.2.0)^39^ with minimum threshold 0.54 for mirrored BAF (mBAF) and minimum 2% rate of heterozygotes per segment.

Clonally related tumors were identified as follows. Each patient’s tumors were stratified by their somatic *MED12* nucleotide change – including a separate strata for *MED12* mutation-negative tumors, if applicable – and each of these strata were then scanned for any matching AI segments. The segments were matched within a 250-Kbp tolerance for both the segment start and end position. Any resulting tumor-pairs with identical *MED12* mutation status and at least one matching AI segment were denoted as clonally related. For patients with clonally related tumors, one tumor was chosen at random and the rest were discarded from statistical analyses.

### Analysis of chromosomal gains and losses through single-nucleotide polymorphism array data

The 2,131 tumors and the respective normal samples were genotyped with Infinium HumanCore-24 (Illumina) SNP-arrays for 309,994 polymorphic loci throughout the whole genome. A total of 11 tumors were discarded from the analysis due to their common clonal origin. Regions displaying allelic imbalance (AI) were identified through genome-wide segmentation of Log R Ratio (LRR) and mirrored B-allele frequency (mBAF) values. Regions of LRR ≤ −0.15 and mBAF≥0.56 denoted regions of allelic loss, and LRR ≥ 0.07 and mBAF≥0.56 denoted gain. The loss and gain regions were used to calculate the total length of AI and to estimate the number of somatic DNA DSBs (or breakpoints). See AI regions in Supplementary Table 1.

### Long-read WGS data processing

Long-read libraries were prepared following the Genomic DNA by Ligation (SQK-LSK109) protocol as per manufacturer’s instructions (Oxford Nanopore Technologies). Sequencing and basecalling was performed on PromethION platform using MinKnow-Live-Basecalling (version 3.4.6). The basecalled reads were aligned with minimap2 (v2.16; *preset: map-ont*) against the GRCh38 reference genome (GCA_000001405.15, excluding alt contigs). Data quality was inspected with NanoStat (v1.1.2) and NanoPlot (v1.20.0). Each aligned long-read library was then processed with Sniffles (v1.0.11; *min_support: 2, min_length: 10, num_reads_report: 2, min_seq_size: 1000*) to identify an initial set of structural variants (SV). The initial SV calls from all tumors and all normals were merged together with SURVIVOR (v1.0.6; *max. 50* *bp distance between breakpoints*) to filter out SVs found in any normal tissue sample (i.e. germline SVs). The remaining SVs constitute somatic events that are likely present only in the tumors. Finally, the somatic SVs were filtered to a minimum 5x alternative allele coverage, minimum 40 bp length deletions and insertions and maximum 9 bp standard deviation for both breakpoint positions (that is, “precise” SVs in Sniffles v1.0.11).

### Determination of tumor DSB number and CCR status

CCR events in 38 whole-genome sequenced (WGS) ULSs – defined as interconnected rearrangements that involve at least three DSBs – were readily available^17^. By assessing the number of DSBs in WGS tumors with and without CCR, and examining the respective SNP-array data, the following criteria for CCR were extrapolated to SNP-array samples (see Supplementary Table 9 for comparison between WGS and SNP-array samples): a single chromosome must have at least four breakpoints or all the chromosomes must have at least six breakpoints. To focus on clonal aberrations, AI regions with mBAF ≥ 0.56, LRR ≤ −0.15 and minimum length of 100,000 bp were considered. Two adjacent regions were considered separate if their difference in LRR was at least 0.1. Whole chromosome losses were discarded. See Fig. 1 A for visualization of these criteria.

To further examine the accuracy (i.e. positive predictive value) of the above criteria, 43 tumors with a putative CCR based on SNP-array analysis, and the paired normal tissue samples, were whole-genome sequenced by long-read DNA sequencing (Oxford Nanopore) to identify somatic DSBs, which were then evaluated for CCR.

The filtered SVs were evaluated for complex chromosomal rearrangements (CCR) as follows. Genome-wide visualizations were produced as circos plots.^40^ A systematic, manual inspection was carried out with the BasePlayer^41^ software (October 2019 version) by visualizing split-read alignments at the observed double-strand breaks (DSB) and identifying interconnected rearrangements similar to^17^. Any rearrangements that involved at least three interconnected DSBs^17^ were classified as a CCR.

### Cell cultures for stretching experiments

Patient-matched uterine leiomyoma and myometrium samples were collected at hysterectomy, to initiate primary cell cultures. The freshly operated uterine leiomyoma and myometrium samples were washed several times with Hanks’ Balanced Salt Solution (HBSS) to remove excess blood, dissected into 1 mm3 pieces with sterile scalpels, and digested overnight at 37 °C with agitation in DMEM containing 2 mg/ml type II collagenase (Life Technologies Inc.), 35 mg/mL amphotericin B (Thermo Fisher Scientific), 1 mg/mL DNase I (Roche, Mannheim, Germany), and 2% (PSG) penicillin/streptomycin/glutamine solution (Thermo Fisher Scientific)^42^. After digestion, cells were filtered through a 50-µm filter (CellTrics) and centrifuged at 146×*g* for 10 min. The cells were then incubated in 2.5 mL of ammonium chloride/potassium (ACK) lysis buffer (Thermo Fisher Scientific) for ~1 min, washed once with HBSS and resuspended in an appropriate volume of DMEM/F12 containing 10% fetal bovine serum and 1x antibiotic-antimycotic (Thermo Fisher Scientific). Isolated cells were assessed for viability by the Trypan Blue exclusion method (Life Technologies Inc.) and counted using the Countess Automated Cell Counter (Life Technologies Inc.). Cells were plated into 25 cm^2^ culture flasks (Corning Inc., Lowell, MA, USA) in DMEM/F12 medium supplemented with 10% fetal bovine serum, antibiotics (100 units/ml penicillin, 100 µg/ml streptomycin), and antimycotic (2 µg/ml amphotericin B), and incubated at 37 °C in a humidified 5% CO2 atmosphere. The initial stage of cell growth was termed passage 0 (P0). When 90% confluent, the cells were subcultured into larger flasks and termed passage 1 (P1). Of note, the primary tumor here was an intramural *MED12* mutation-positive tumor (c.131 G > A, p.G44D), and the cells derived from the tumor kept the same mutation.

### Stretching and immunofluorescence

Low passage (passage 2–6) leiomyoma and myometrium cells were plated at a density of 75,000 cells/well in flexible-bottomed six-well culture plates (BioFlex® Culture Plates, Dunn Labortechnik GmbH) coated with collagen type I in 3 ml of complete serum-containing growth medium. After 24 h, the medium was replaced with serum-free medium and cells were serum-starved for 12 h prior to stretching to synchronize cell cycle. The cells were then returned to complete serum-containing medium for 3 h before starting the stretching and maintained in this medium until experiment termination. Static mechanical stretch with 26% elongation was applied for 24–48 h using Flexcell FX-5000 Tension System (Flexcell® International Corp., PA), followed by cell fixation immediately or after a recovery period. Control cells (referred to as “non-stretched cells”) were plated on the same flexible-bottomed culture plates, but were not stretched. In parallel experiments serving as a positive control for DNA damage, leiomyoma and myometrium cells from the same cultures were plated on glass coverslips in regular six-well culture plates and received a 5 Gy dose of ionizing irradiation, followed by a 6 h recovery period. Similar to the stretching experiments, the cells were serum-starved overnight and then returned to complete serum-containing medium before irradiation.

After the treatments, the cells were washed with PBS, pH 7.4 and fixed with 2% paraformaldehyde for 10 min at 4 °C. After fixation, the silicone rubber membranes were cut into multiple pieces for multiple staining, and transferred into a 24-well plate. Cells were permeabilized with 0.2% Triton X-100 for 20 min, blocked, and incubated overnight at 4 °C with primary antibodies listed in Supplementary Table 10. Cells were then incubated with fluorescently labelled secondary antibodies (goat anti-mouse Alexa 488, Invitrogen A11029, diluted 1:1000; goat anti-rabbit Alexa 647, Invitrogen A21245, diluted 1:1000), counterstained with 2 μg/mL of Hoechst and mounted on glass slides with ProLong Gold Antifade Mountant (Invitrogen). Indirect immunofluorescence against γH2Ax was performed on two sets of membranes: as a double staining together with Ki67, and as a double staining with 53BP1. RAD51 was stained together with CyclinA2. cCasp3 and p14ARF were stained together with 488-Phalloidin. Images were acquired with an Opera Phenix high-content confocal microscope equipped with a 5x pre-scan air objective (NA 0.16) and a ×40 re-scan air objective (NA 0.6, working distance 3.28 mm, depth of focus 3.3 µm, Zeiss) and four excitation lasers (405 nm with emission band-pass filter 435/480; ex 488, em 500/550; ex 561, em 570/630; ex 647, em 650/760). Selected objects were imaged with 10 predetermined Z focus planes with laser-based autofocusing. The images were captured with two Andor Zyla sCMOS cameras (16 bit, field of view 650 × 650 μm^2^, effective xy resolution 0.66 µm). Image analysis was performed with Harmony 4.9 software at FIMM High Content Imaging and Analysis Unit. Harmony software parameters utilized in the image analysis are enumerated in Supplementary Data 3.

The γH2Ax, 53BP1, RAD51, and Ki67 stainings were quantified as the number of foci per nucleus, cleaved caspase-3 using combined nuclear and perinuclear intensity, and the remaining markers using nuclear intensity alone. Nuclei with >10 γH2Ax foci were counted as positive for DNA damage. Nuclei with both >5 53BP1 foci and >10 γH2Ax foci were counted as positive for NHEJ repair. CyclinA2-positivity was assessed based on nuclear intensity; Supplementary Data 4 gives both the quantified intensity values and resulting CyclinA2-positivity estimates. All CyclinA2-positive nuclei with >5 RAD51 foci were counted as positive for HR repair, and the rest of the CyclinA2-positive nuclei as negative for HR repair. Nuclei with >0 Ki67 foci were counted as positive for proliferation. cCasp3-positivity was thresholded at the 3rd quartile of combined nuclear and perinuclear cCasp3 intensity, calculated from non-stretched myometrium and myoma control cells separately. p14ARF-positivity was assessed based on nuclear intensity (Supplementary Data 4). All reported percentages (“% of positive nuclei”) were calculated with respect to total numbers of nuclei, except for RAD51-positivity, which was calculated among CyclinA2-positive nuclei. Computational estimates of nuclear area (µm²) and roundness of the nuclei (Harmony software; range 0–1, where values close to 1 are more round) were quantified for all nuclei identified in the image analysis.

### Statistics and reproducibility

Parity information was available for 1946 SNP-array tumors from 628 patients. 11 tumors were clonally related and removed from the analysis. The average for breakpoint number, total length of loss regions and total length of gain regions are reported for tumors. Because of the non-normality of the distributions, 95% percentile confidence interval of the mean was calculated by bootstrapping using 1000 bootstrap replicates with R package boot (v.1.3–20)^43^. The association between allelic imbalance and parity was assessed by fitting a generalized estimating equations (GEE) model using the geeglm function from the R package geepack (v.1.2-1)^44^. The GEE method was chosen because multiple tumors from the same patient can be dependent on each other. Exchangeable correlation structure was assumed and a robust sandwich estimator for variance is implemented in the model. In two separate analyses, CCR status and whole chromosome events were modeled assuming binomial random component and logit link function. The total length of AI regions was modeled using Poisson random component and log link function.

In addition to full data, *MED12* mutated and wild-type (WT) tumors were also considered separately because the subgroups differ in their biological properties^15^. Confidence intervals (CI) and *P*-values were Bonferroni corrected (adjusted alpha 0.05/11). Confounding variables for age, use of oral contraceptives, menopause status, and smoking status were included. In addition, tumor location (intramural, submucous, subserous) and the size of the tumors were studied in relation to total length of AI.

The effects of mechanical stretching were studied in a single multi time point experiment performed in parallel for primary myometrium and leiomyoma cells in duplicate culture wells per experimental condition. Effects of mechanical stretching on these two cell types were assessed with two complementary approaches: first, all time points combined and, second, each time point separately. For the combined analysis, difference in marker-positivity between stretched cells and non-stretched control cells was assessed with logistic regression, adjusted for categorical covariates on the three time points (24 h, 30 h and 48 h) and, for γH2Ax, the two sets of double staining (Ki67 and 53BP1). Goodness-of-fit and residual diagnostics were inspected with the DHARMa package (v0.4.1). Effect size estimates from logistic regression are reported as odds-ratios (OR) of marker-positivity in stretched compared to control cells, together with a 95% CI and significance level (P). Multiple-test adjusted *P*-values were calculated over DNA damage/repair tests (Holm’s method; Supplementary Table 6). For the time-point specific analysis, Fisher’s exact test *P*-values were computed to assess differences in marker-positivity at each time point. For visualization, we show proportions (scale 0–100%) of marker-positive nuclei and their fold-change between stretched and control cells at each time-point. Fold-change estimates include a pseudocount of +1, and the respective 95% CI of fold-changes were calculated by bootstrapping (10,000 samples). Multiple-test adjusted *P*-values were calculated over DNA damage/repair tests (Holm’s method; Supplementary Table 7).

The possible enrichment of ULMS in multiparous women was investigated by applying conditional logistic regression to the population-based registry data. We modeled a dose-response relationship between disease outcome and number of children in those cases and controls who had given birth at least once, by having the number of children as the predictor and disease status as the response in conditional logistic regression, stratifying by each matched case-control set. Regression analysis was done in R, using the clogit function provided in R package survival (version 2.38).

The power of our registry analysis was calculated with the epiR package. We used Statistics Finland’s (SF) data describing number of deliveries by mother’s birth year to estimate the birth count distribution in our data. Because SF data groups together all birth counts of four or above, we used this as our definition of exposure to a large number of births in the power analysis. As most of the parous patients in our data were born before 1940, in which group higher numbers of deliveries were common, we estimated exposure prevalence to be 15% in the parous background population. With 399 cases and 2,056 controls, an odds ratio of ~1.50 for leiomyosarcoma in the exposed group would reach a power of 0.8 in the analysis. The power calculations were done with R package epiR (version 1.0–4).

### Reporting summary

Further information on research design is available in the Nature Research Reporting Summary linked to this article.

## Supplementary information

Supplementary Information

Description of Additional Supplementary Files

Supplementary Data 1

Supplementary Data 2

Supplementary Data 3

Supplementary Data 4

Reporting Summary

## Data Availability

The data that support this study are available from the corresponding author upon reasonable request. The somatic allelic imbalance segment data generated in this study are provided in Supplementary Data 1. The tumor- and patient-specific variables used in this study are provided in Supplementary Data 2. The Harmony software parameters used in this study are provided in Supplementary Data 3. The quantified imaging data generated in this study are provided in Supplementary Data 4. The GRCh38 reference genome data used in this study are available in the GenBank database under accession code GCA_000001405.15. Source data are provided with this paper.

## Source data

Source Data

## References

[1] Hatch EM, Fischer AH, Deerinck TJ, Hetzer MW (2013). Catastrophic nuclear envelope collapse in cancer cell micronuclei. Cell.

[2] Lim S, Quinton RJ, Ganem NJ (2016). Nuclear envelope rupture drives genome instability in cancer. Mol. Biol. Cell.

[3] Shah P, Wolf K, Lammerding J (2017). Bursting the Bubble - Nuclear Envelope Rupture as a Path to Genomic Instability?. Trends Cell Biol..

[4] Cho S (2019). Mechanosensing by the Lamina Protects against Nuclear Rupture, DNA Damage, and Cell-Cycle Arrest. Dev. Cell.

[5] Xia Y (2018). Nuclear rupture at sites of high curvature compromises retention of DNA repair factors. J. Cell Biol..

[6] Hatch EM (2018). Nuclear envelope rupture: little holes, big openings. Curr. Opin. Cell Biol..

[7] Denais CM (2016). Nuclear envelope rupture and repair during cancer cell migration. Science.

[8] Irianto J (2016). Nuclear constriction segregates mobile nuclear proteins away from chromatin. Mol. Biol. Cell.

[9] Shah P (2021). Nuclear Deformation Causes DNA Damage by Increasing Replication Stress. Curr. Biol..

[10] Fröhling S, Döhner H (2008). Chromosomal abnormalities in cancer. N. Engl. J. Med..

[11] Rehman KS, Yin S, Mayhew BA, Word RA, Rainey WE (2003). Human myometrial adaptation to pregnancy: cDNA microarray gene expression profiling of myometrium from non-pregnant and pregnant women. Mol. Hum. Reprod..

[12] Smith R, Imtiaz M, Banney D, Paul JW, Young RC (2015). Why the heart is like an orchestra and the uterus is like a soccer crowd. Am. J. Obset. Gynecol..

[13] Baird DD, Dunson DB, Hill MC, Cousins D, Schectman JM (2003). High cumulative incidence of uterine leiomyoma in black and white women: ultrasound evidence. Am. J. Obstet. Gynecol..

[14] Stewart EA (2001). Uterine fibroids. Lancet.

[15] Mehine M (2016). Integrated data analysis reveals uterine leiomyoma subtypes with distinct driver pathways and biomarkers. Proc. Natl Acad. Sci. U. S. A..

[16] Mehine M, Mäkinen N, Heinonen H-R, Aaltonen LA, Vahteristo P (2014). Genomics of uterine leiomyomas: insights from high-throughput sequencing. Fertil. Steril..

[17] Mehine M (2013). Characterization of Uterine Leiomyomas by Whole-Genome Sequencing. N. Eng. J. Med..

[18] Liegl-Atzwanger B (2016). Exploring chromosomal abnormalities and genetic changes in uterine smooth muscle tumors. Mod. Pathol..

[19] Berta, D. G. *et al*. Deficient H2A.Z deposition is associated with genesis of uterine leiomyoma. *Nature*10.1038/s41586-021-03747-1 (2021). 10.1038/s41586-021-03747-1 34349258

[20] Luijten MNH, Lee JXT, Crasta KC (2018). Mutational game changer: Chromothripsis and its emerging relevance to cancer. Mutat. Res..

[21] Ly P, Cleveland DW (2017). Rebuilding Chromosomes After Catastrophe: Emerging Mechanisms of Chromothripsis. Trends Cell Biol..

[22] Chudasama P (2018). Integrative genomic and transcriptomic analysis of leiomyosarcoma. Nat. Commun..

[23] Yamaguchi M (2019). Case of leiomyosarcoma arising from subserosal leiomyoma. J. Obstet. Gynaecol. Res..

[24] Raab M (2016). ESCRT III repairs nuclear envelope ruptures during cell migration to limit DNA damage and cell death. Science.

[25] Hieronymus H (2014). Copy number alteration burden predicts prostate cancer relapse. Proc. Natl Acad. Sci. U. S. A..

[26] Lalonde E (2014). Tumour genomic and microenvironmental heterogeneity for integrated prediction of 5-year biochemical recurrence of prostate cancer: a retrospective cohort study. Lancet Oncol..

[27] Bayani J (2008). Distinct patterns of structural and numerical chromosomal instability characterize sporadic ovarian cancer. Neoplasia.

[28] Feng C (2018). Cyclic mechanical tension reinforces DNA damage and activates the p53-p21-Rb pathway to induce premature senescence of nucleus pulposus cells. Int. J. Mol. Med..

[29] Mao Z, Bozzella M, Seluanov A, Gorbunova V (2008). Comparison of nonhomologous end joining and homologous recombination in human cells. DNA Repair.

[30] Khanna KK, Jackson SP (2001). DNA double-strand breaks: signaling, repair and the cancer connection. Nat. Genet..

[31] Sharma, S., Raghavan, S. C. *Nonhomologous DNA End Joining*. *Encyclopedia of Cell Biology*. *Volume 1*. (Academic Press, Cambridge, 2016).

[32] Capo-Chichi CD, Yeasky TM, Smith ER, Xu X-X (2016). Nuclear envelope structural defect underlies the main cause of aneuploidy in ovarian carcinogenesis. BMC Cell Biol..

[33] Alotaibi M, Arrowsmith S, Wray S (2015). Hypoxia-induced force increase (HIFI) is a novel mechanism underlying the strengthening of labor contractions, produced by hypoxic stresses. Proc. Natl Acad. Sci. U. S. A..

[34] Heinonen H-R (2014). MED12 mutation frequency in unselected sporadic uterine leiomyomas. Fertil. Steril..

[35] Heinonen H-R (2017). Multiple clinical characteristics separate MED12-mutation-positive and -negative uterine leiomyomas. Sci. Rep..

[36] Mäkinen N (2011). MED12, the mediator complex subunit 12 gene, is mutated at high frequency in uterine leiomyomas. Science.

[37] Leinonen MK, Miettinen J, Heikkinen S, Pitkäniemi J, Malila N (2017). Quality measures of the population-based Finnish Cancer Registry indicate sound data quality for solid malignant tumours. Eur. J. Cancer.

[38] Diskin SJ (2008). Adjustment of genomic waves in signal intensities from whole-genome SNP genotyping platforms. Nucleic Acids Res..

[39] Staaf J (2008). Segmentation-based detection of allelic imbalance and loss-of-heterozygosity in cancer cells using whole genome SNP arrays. Genome Biol..

[40] Gu Z, Gu L, Eils R, Schlesner M, Brors B (2014). circlize implements and enhances circular visualization in R. Bioinformatics.

[41] Katainen R (2018). Discovery of potential causative mutations in human coding and noncoding genome with the interactive software BasePlayer. Nat. Protoc..

[42] Chang HL (2010). Uterine leiomyomas exhibit fewer stem/progenitor cell characteristics when compared with corresponding normal myometrium. Reprod. Sci..

[43] Davison, A. C. & Hinkley, D. V. *Bootstrap Methods and Their Application*. (Cambridge University Press, Cambridge, 1997).

[44] Halekoh, U., Højsgaard, S. & Yan, J. The R Package geepack for Generalized Estimating Equations. *J. Stat. Softw*. **15**, (2006).

