# Supplementary material for: Parity associates with chromosomal damage in uterine leiomyomas
Source: Nat Commun. 2021 Sep 14;12:5448. doi: 10.1038/s41467-021-25806-x (PMC8440576; doi:10.1038/s41467-021-25806-x)
Supplement: Supplementary file 1 — Supplementary Information [file 41467_2021_25806_MOESM1_ESM.pdf]

Supplementary Information

Parity Associates With Chromosomal Damage in Uterine Leiomyomas

SUPPLEMENTARY FIGURES **3**

Supplementary Fig. 1: Association test between total length of AI and parity in all tumors 3

Supplementary Fig. 2: Association test between total length of AI and parity in MED12 tumors 4

Supplementary Fig. 3: Association test between total length of AI and parity in WT tumors 5

Supplementary Fig. 4: Association test between whole-chromosome losses and parity in WT tumors 6

Supplementary Fig. 5: Circos plots of somatic chromosomal rearrangements 12

Supplementary Fig. 6: Association test between CCR and parity in all tumors 14

Supplementary Fig. 7: Association test between CCR and parity in MED12 tumors 15

Supplementary Fig. 8: Association test between CCR and parity in WT tumors 16

Supplementary Fig. 9: Relationship between tumor location and total loss in MED12 tumors 17

Supplementary Fig. 10: Numbers of nuclei identified in fluorescence staining image analysis 18

Supplementary Fig. 11: Summary of ionizing irradiation experiments 19

Supplementary Fig. 12: Summary of γH2Ax staining 21

Supplementary Fig. 13: Summary of 53BP1 staining 22

Supplementary Fig. 14: Summary of RAD51 staining 23

Supplementary Fig. 15: Representative images of γH2Ax, Ki67, CyclinA2 and RAD51 positivity 24

Supplementary Fig. 16: Summary of cCasp3 staining 25

Supplementary Fig. 17: Summary of p14ARF staining 26

Supplementary Fig. 18: Summary of Ki67 staining 27

Supplementary Fig. 19: Summary of CyclinA2 staining 28

Supplementary Fig. 20: In silico estimates of differences in nuclear morphology 29

SUPPLEMENTARY TABLES **31**

Supplementary Table 1: Patient characteristics 31

Supplementary Table 2. Allelic imbalance characteristics of ULs (N=1935) 32

Supplementary Table 3. Confirmation of CCR by long-read whole-genome sequencing 33

Supplementary Table 4. The effect of tumor location to allelic imbalance 35

Supplementary Table 5. Numbers of nuclei identified in fluorescence staining image analysis 36

Supplementary Table 6. DNA damage and repair in stretched and non-stretched cells 37

Supplementary Table 7. DNA damage and repair in each time point 38
Supplementary Table 8. Summary of other markers in stretched and non-stretched cells 39
Supplementary Table 9. Comparison of CCR status between WGS and SNP-array 41
Supplementary Table 10. List of antibodies 42

SUPPLEMENTARY FIGURES

```
geeglm(formula = total ~ age + smoking + hormonal + factor(mp) +
     para, family = poisson, data = data, id = id, corstr =
"exchangeable")

 Coefficients:
           Estimate Std.err   Wald Pr(>|W|)
(Intercept) 17.4403  0.7899 487.53  < 2e-16 ***
age         -0.0228  0.0181   1.59     0.21
smoking      0.1613  0.1849   0.76     0.38
hormonal     0.2046  0.1678   1.49     0.22
mpHRT        0.2865  0.3252   0.78     0.38
mpPost       0.6904  0.5320   1.68     0.19
para         0.2798  0.0487  32.99  9.3e-09 ***
---
Signif. codes:  0 '***' 0.001 '**' 0.01 '*' 0.05 '.' 0.1 ' ' 1

Estimated Scale Parameters:
           Estimate  Std.err
(Intercept) 1.96e+08 31074287

Correlation: Structure = exchangeable  Link = identity

Estimated Correlation Parameters:
     Estimate Std.err
alpha   0.0376 0.00972
Number of clusters:   596   Maximum cluster size: 19
```

Supplementary Fig. 1: Association test between total length of AI and parity in all tumors

```
Call:
geeglm(formula = total ~ age + smoking + hormonal + factor(mp) +
     para, family = poisson, data = med12, id = id, corstr =
"exchangeable")

 Coefficients:
           Estimate  Std.err   Wald Pr(>|W|)
(Intercept) 18.12104  0.81418 495.36   <2e-16 ***
age         -0.05413  0.01834   8.71   0.0032 **
smoking      0.00737  0.22462   0.00   0.9738
hormonal    -0.26151  0.21265   1.51   0.2188
factor(mp)1  0.51123  0.40276   1.61   0.2043
factor(mp)2  0.37854  0.48284   0.61   0.4330
para         0.33252  0.09917  11.24   0.0008 ***
---
Signif. codes:  0 '***' 0.001 '**' 0.01 '*' 0.05 '.' 0.1 ' ' 1

Estimated Scale Parameters:
           Estimate  Std.err
(Intercept) 98124901 18059698

Correlation: Structure = exchangeable  Link = identity

Estimated Correlation Parameters:
     Estimate Std.err
alpha -0.00518 0.00788
Number of clusters:   429   Maximum cluster size: 19
```

Supplementary Fig. 2: Association test between total length of AI and parity in *MED12* tumors

```
geeglm(formula = total ~ age + smoking + hormonal + factor(mp) +
     para, family = poisson, data = wt, id = id, corstr =
"exchangeable")

 Coefficients:
             Estimate   Std.err   Wald Pr(>|W|)
(Intercept) 17.823628  0.791114 507.59   <2e-16 ***
age         -0.000772  0.017248   0.00   0.9643
smoking      0.030500  0.198564   0.02   0.8779
hormonal     0.486747  0.173924   7.83   0.0051 **
mpHRT       -0.101283  0.343116   0.09   0.7679
mpPost       0.331938  0.471526   0.50   0.4815
para         0.047196  0.053548   0.78   0.3781
---
Signif. codes:  0 '***' 0.001 '**' 0.01 '*' 0.05 '.' 0.1 ' ' 1

Estimated Scale Parameters:
           Estimate  Std.err
(Intercept) 2.01e+08 35927185

Correlation: Structure = exchangeable  Link = identity

Estimated Correlation Parameters:
     Estimate Std.err
alpha 0.209   0.0477
Number of clusters:   306   Maximum cluster size: 1
```

Supplementary Fig. 3: Association test between total length of AI and parity in WT tumors

```
geeglm(formula = wg ~ age + smoking + mp + combined + para, family
= binomial,
     data = data, id = patient, corstr = "exchangeable")

 Coefficients:
            Estimate  Std.err   Wald Pr(>|W|)
(Intercept) -3.06794  1.97459  2.414   0.1203
age         -0.01238  0.03182  0.151   0.6973
smoking     -0.41448  0.43926  0.890   0.3454
mppost       1.71932  0.69512  6.118   0.0134 *
mppre       -0.17527  0.77149  0.052   0.8203
combined     0.40718  0.39354  1.071   0.3008
para         0.51527  0.13021 15.660 7.58e-05 ***
---
Signif. codes:  0 '***' 0.001 '**' 0.01 '*' 0.05 '.' 0.1 ' ' 1

Estimated Scale Parameters:
           Estimate Std.err
(Intercept)   0.8443  0.4793

Correlation: Structure = exchangeable  Link = identity

Estimated Correlation Parameters:
     Estimate Std.err
alpha  0.05363 0.03363
```

Supplementary Fig. 4: Association test between whole-chromosome losses and parity in WT tumors

My_6290.1T3

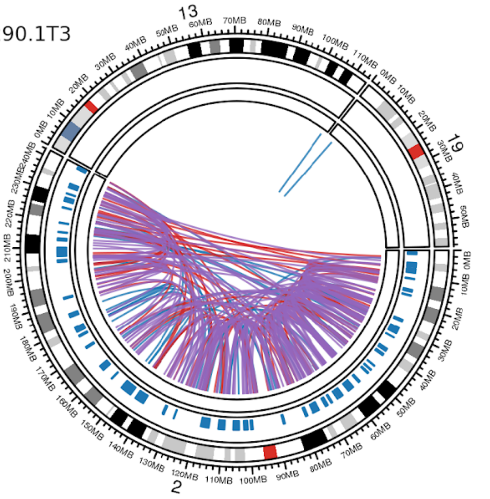

My_6223.1T1

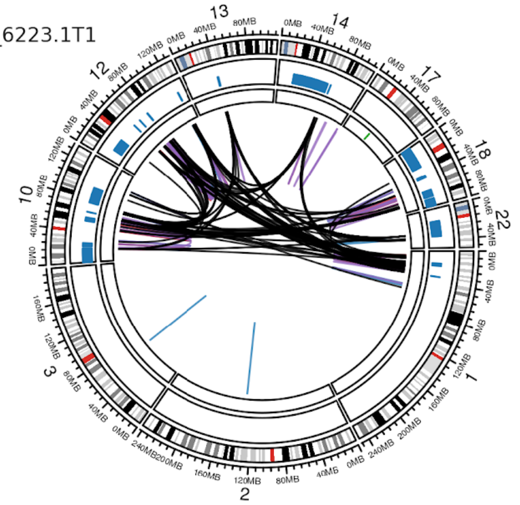

My_6124.1T1

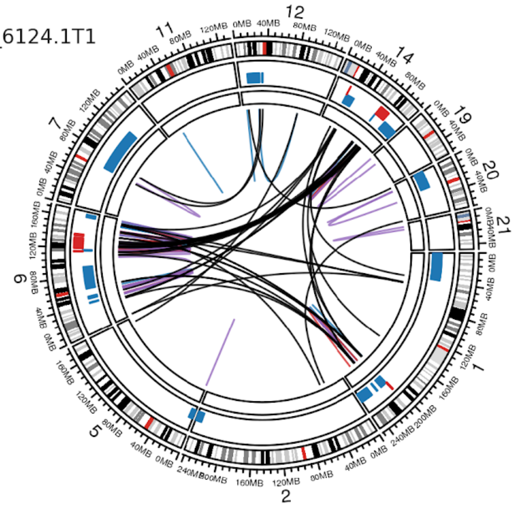

My_6107.1T1

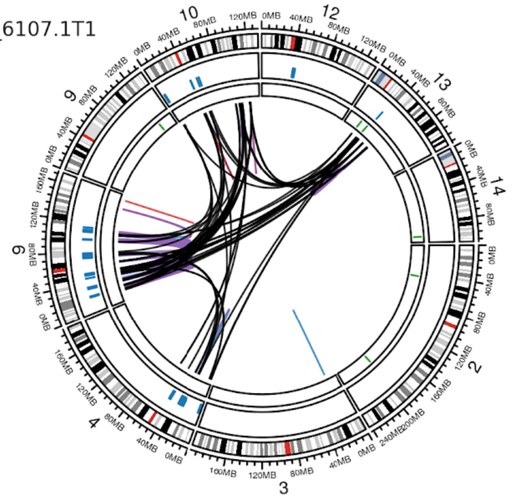

My_8018.1T1

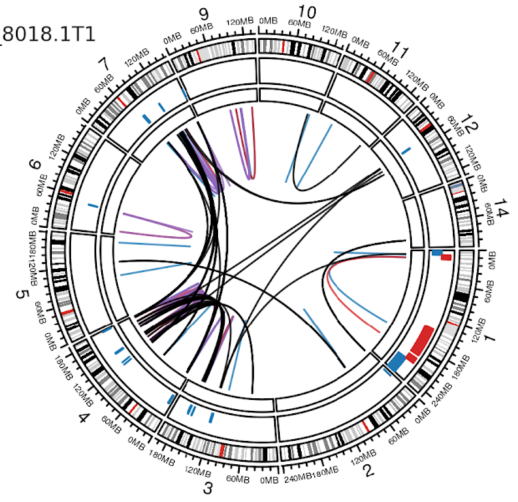

My_6322.1T1

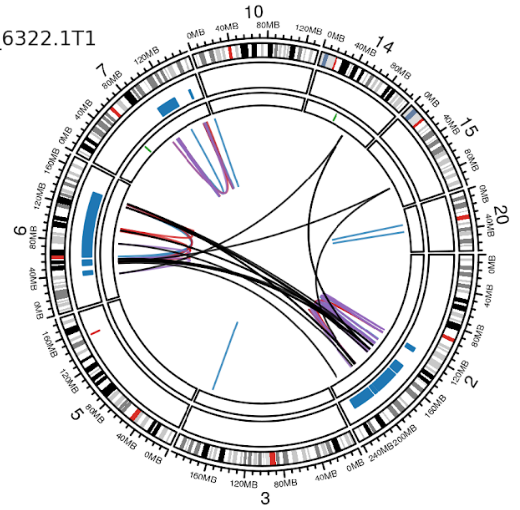

My_6310.1T1

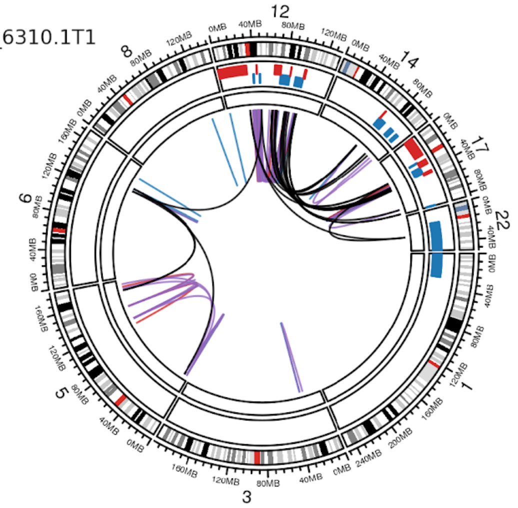

My_6306.1T1

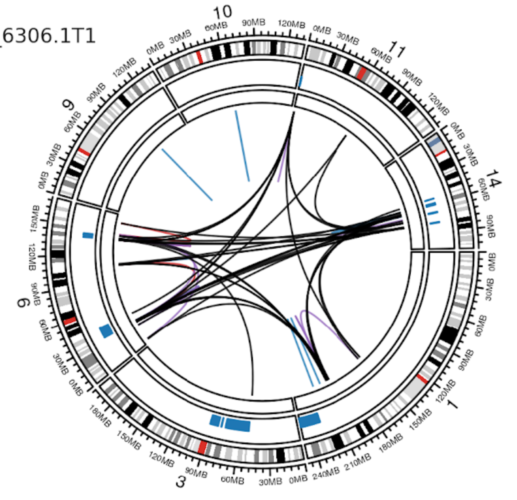

My_6037.1T1

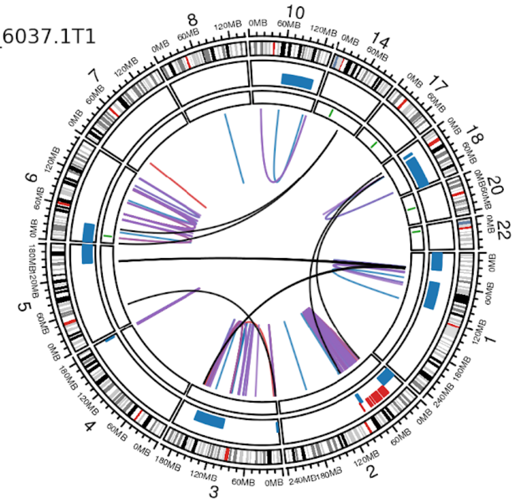

My_6250.1T1

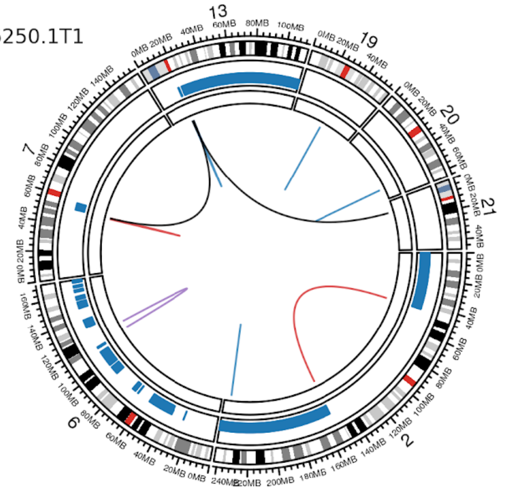

My_6211.1T2

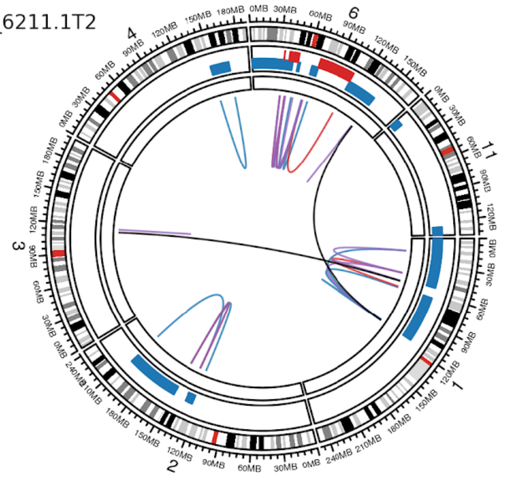

My_6056.1T1

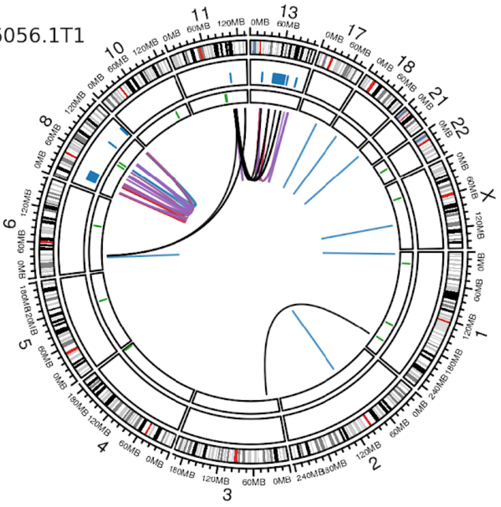

My_6022.1T1

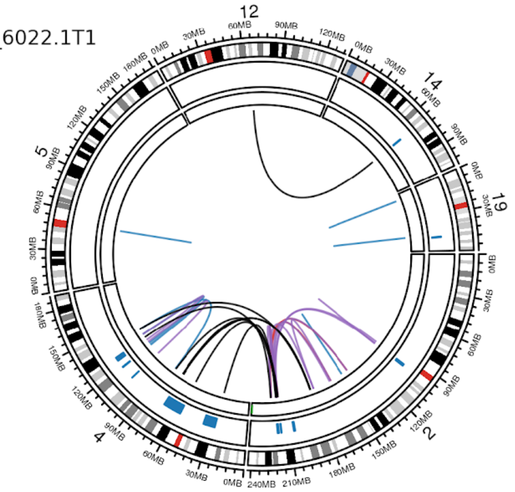

My_6110.1T1

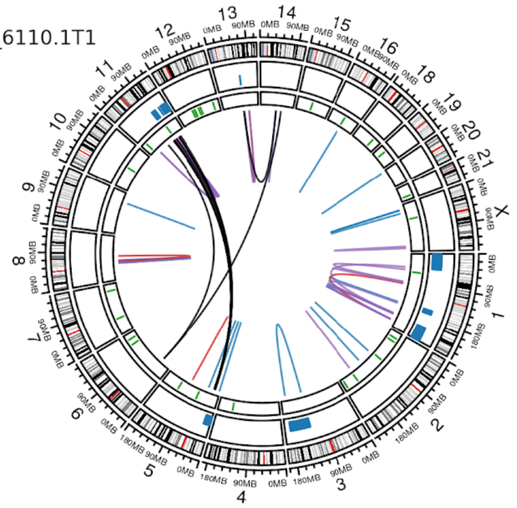

My_6091.1T1

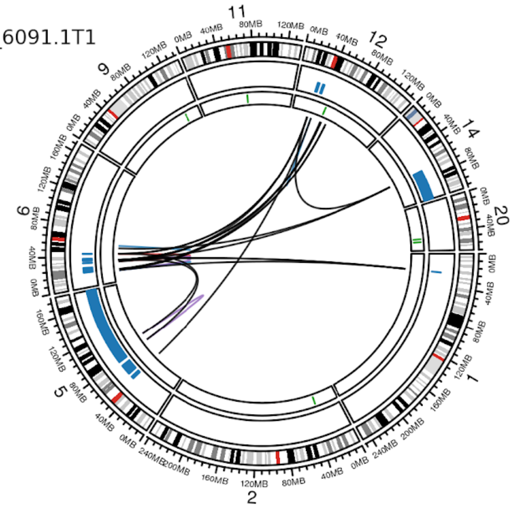

My_6011.1T1

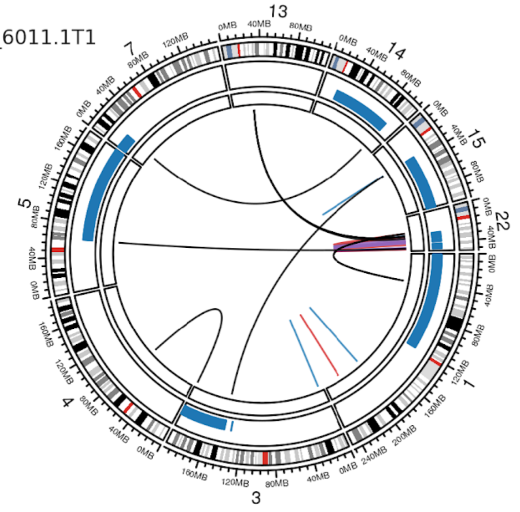

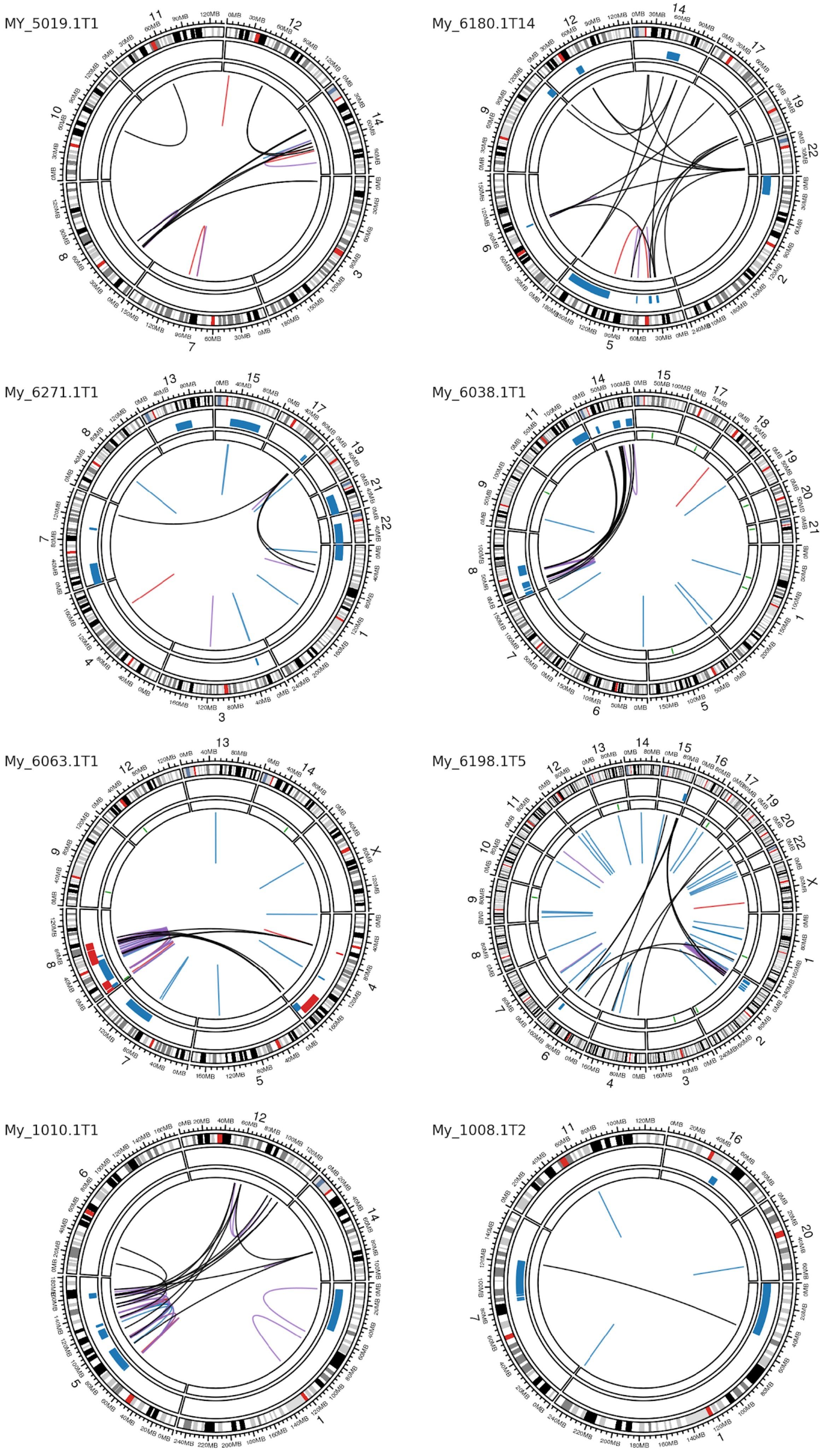
MY_5019.1T1
My_6180.1T14
My_6271.1T1
My_6038.1T1
My_6063.1T1
My_6198.1T5
My_1010.1T1
My_1008.1T2

My_6191.1T1

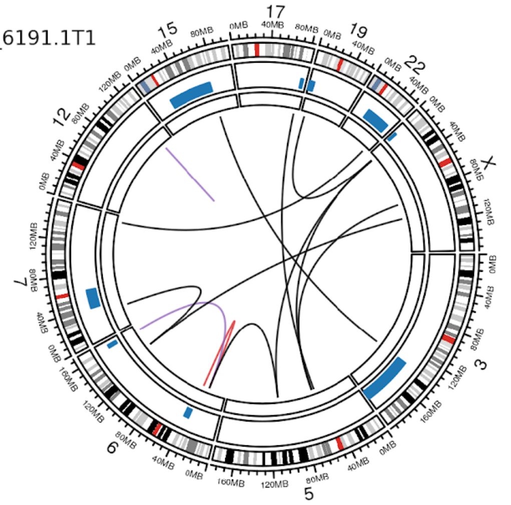

My_8016.1T1

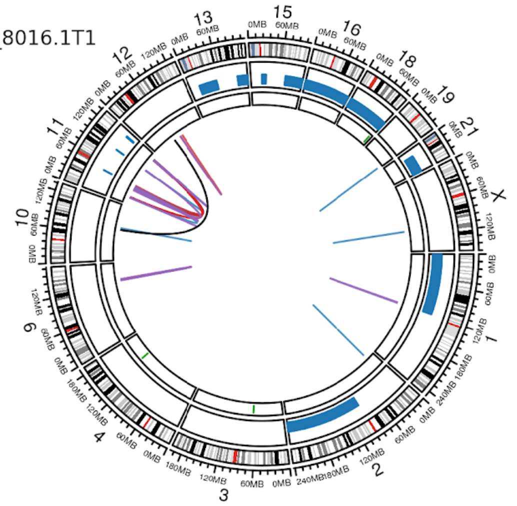

My_6168.1T3

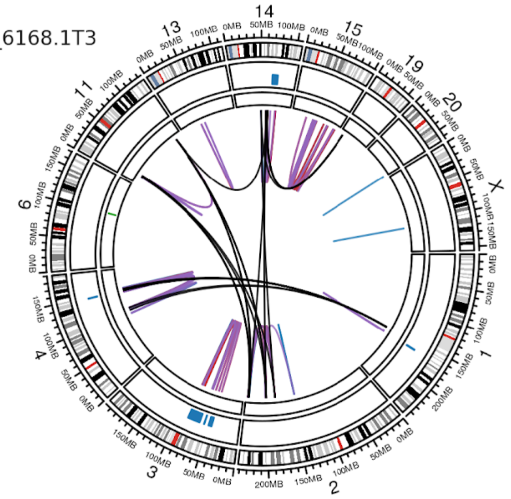

My_6161.1T8

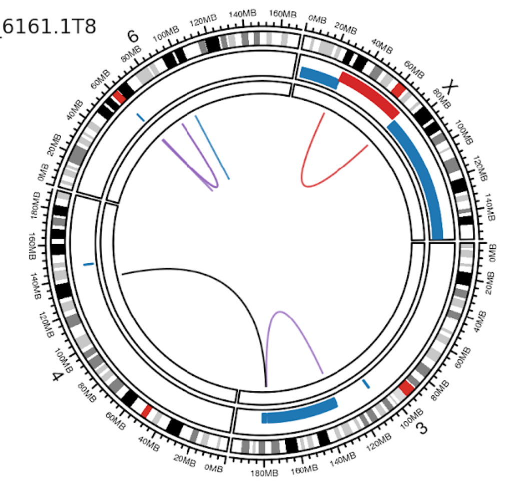

My_6033.1T1

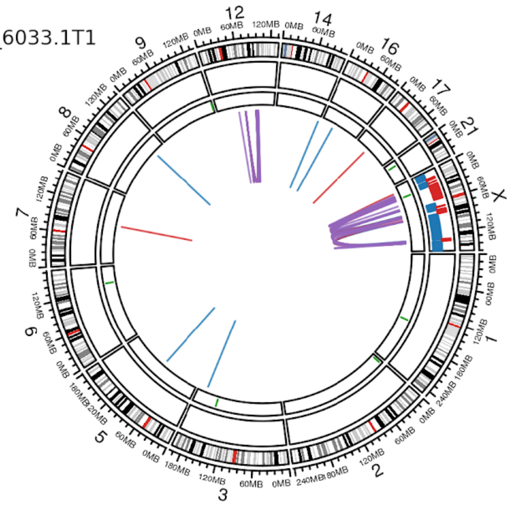

My_6286.1T1

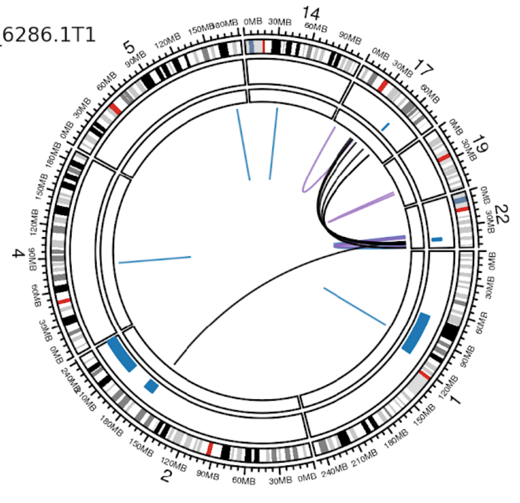

My_6191.1T3

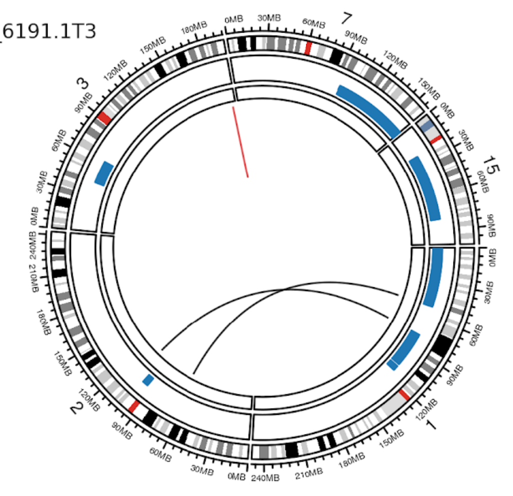

My_6013.1T3

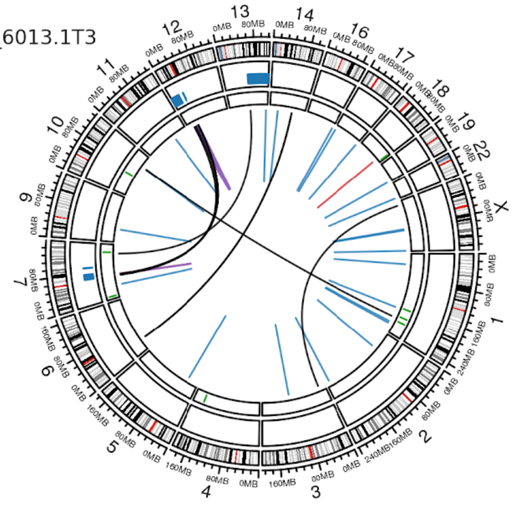

My_8016.1T2

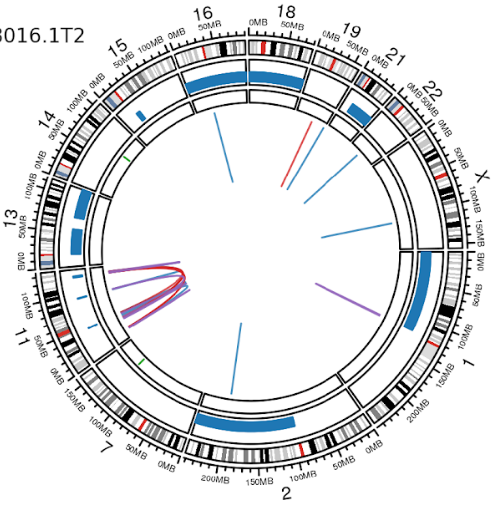

My_6447.1T1

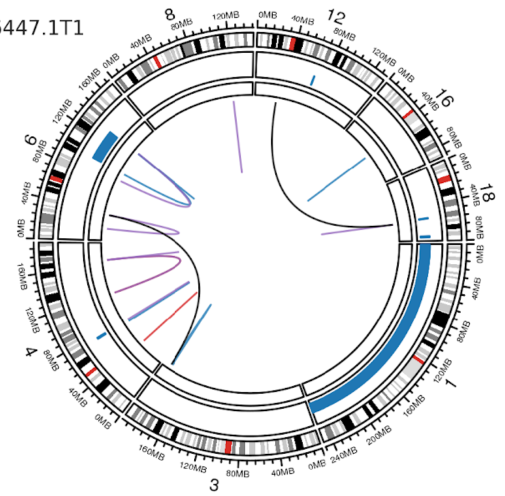

My_6334.1T2

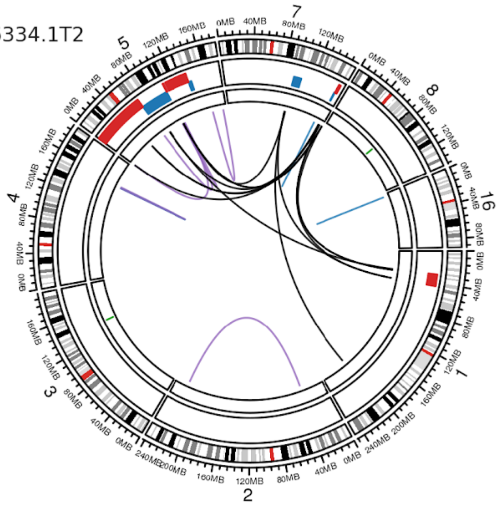

My_6125.1T1

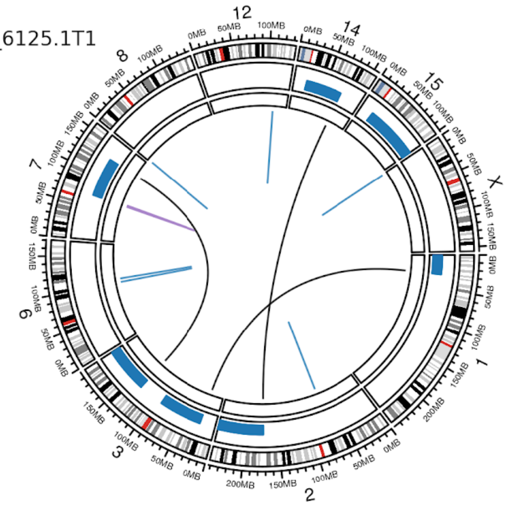

My_6125.1T2

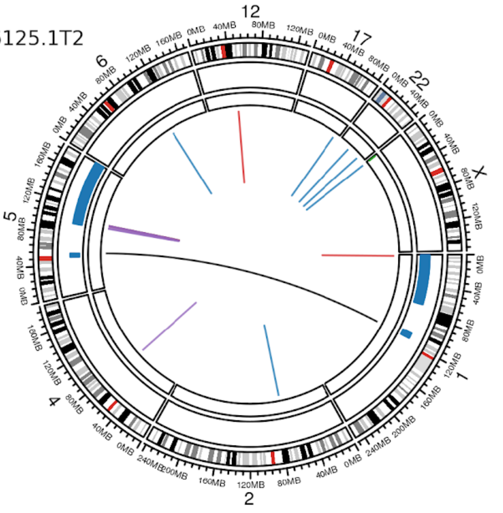

My_6244.1T2

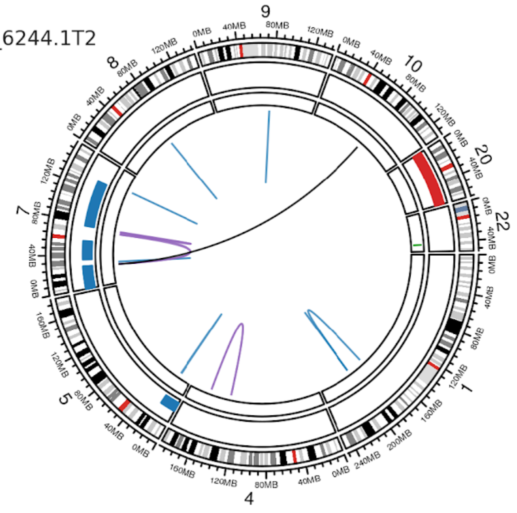

My_6338.1T1

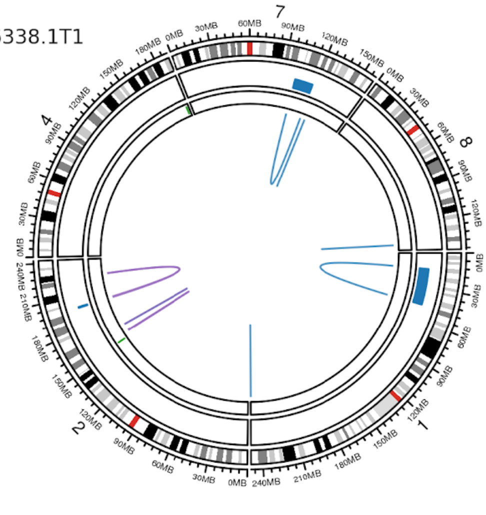

My_6303.1T1

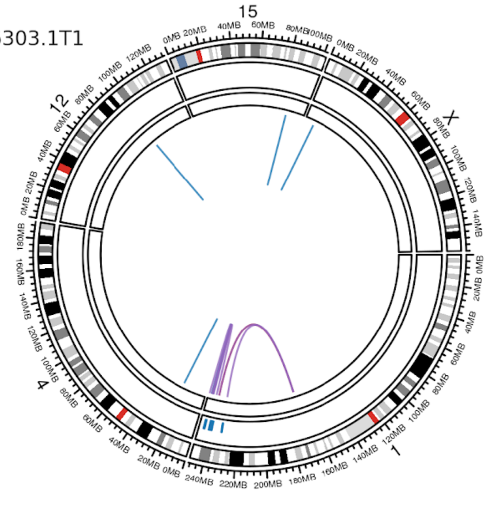

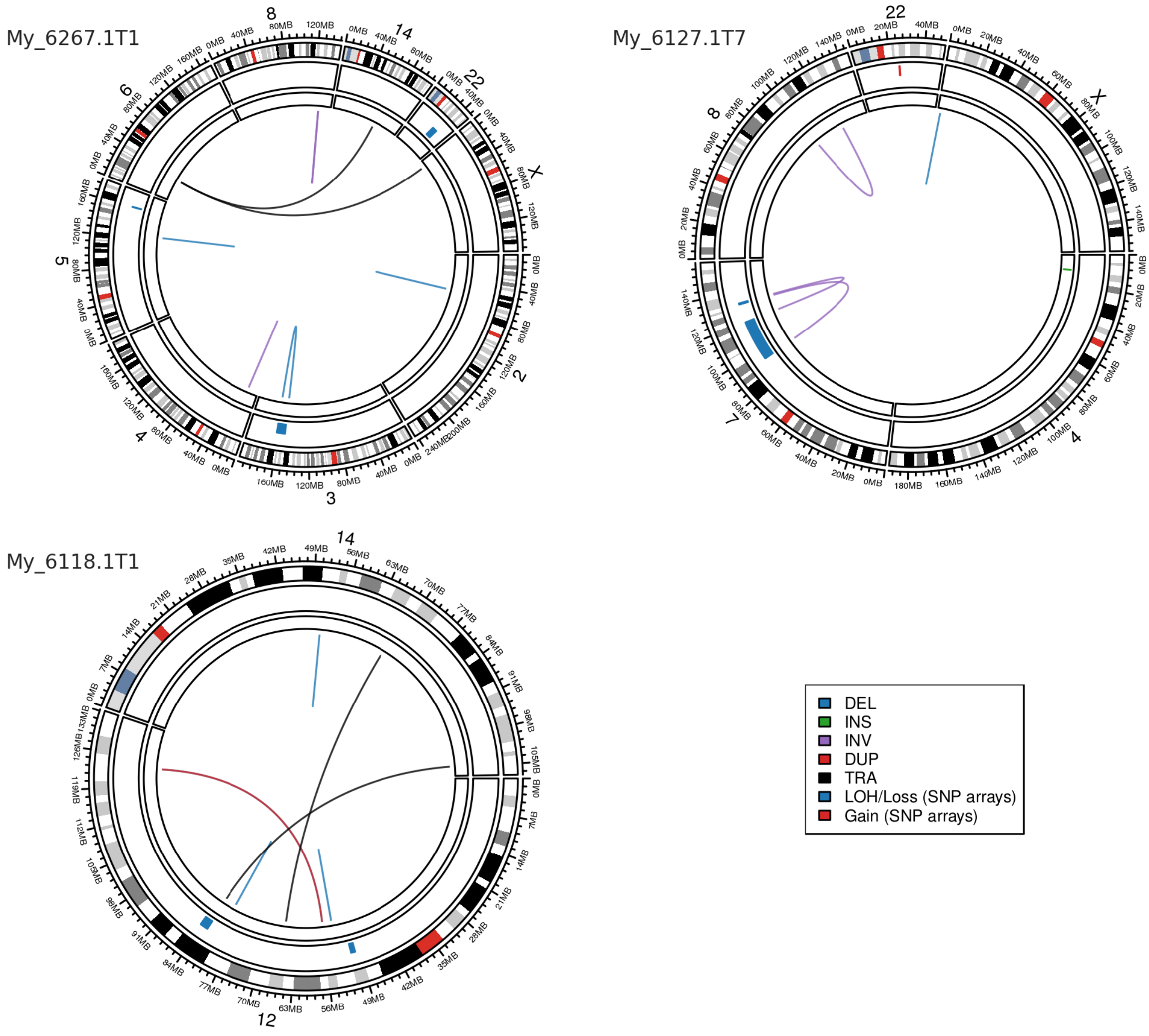

Supplementary Fig. 5: Circos plots of somatic chromosomal rearrangements

A circos plot for each of the 43 long-read sequenced tumors. The order of the plots follows Supplementary Table 4, and each plot displays the following annotations. The outer annotation track shows all chromosomes affected by somatic aberrations (centromeres are red; grayscale for cytobands). The next annotation track shows the SNP-array based AI regions (loss regions are blue; gain red). The inner annotation track shows the insertion

positions (green). The colored arches show intra-chromosomal events (deletions are blue; inversions violet; duplications red) and inter-chromosomal translocations (black). Due to the limited resolution, some visual elements may overlap and, thus, appear contiguous. Two tumors, My_8016.1T1 and My_8016.1T2, are clonally related.

```
geeglm(formula = CCR ~ age + smoking + hormonal + factor(mp) +
     para, family = binomial, data = data, id = id, corstr =
"exchangeable")

 Coefficients:
           Estimate Std.err  Wald Pr(>|W|)
(Intercept) -1.3409  0.7600  3.11    0.078 .
age         -0.0234  0.0165  2.03    0.154
smoking     -0.1794  0.1868  0.92    0.337
hormonal    -0.2340  0.1718  1.85    0.173
mpHRT        0.2233  0.3699  0.36    0.546
mpPost       0.7253  0.3723  3.79    0.051 .
para         0.2542  0.0584 18.92   1.4e-05 ***
---
Signif. codes:  0 '***' 0.001 '**' 0.01 '*' 0.05 '.' 0.1 ' ' 1

Estimated Scale Parameters:
           Estimate Std.err
(Intercept)     0.955   0.171

Correlation: Structure = exchangeable  Link = identity

Estimated Correlation Parameters:
     Estimate Std.err
alpha   0.0235  0.0131
Number of clusters:   596   Maximum cluster size: 19
```

Supplementary Fig. 6: Association test between CCR and parity in all tumors

```
geeglm(formula = CCR ~ age + smoking + mp + combined + para,
     family = "binomial", data = data, id = data$patient, corstr =
"exchangeable")

 Coefficients:
           Estimate Std.err  Wald Pr(>|W|)
(Intercept) -1.8530  1.4433  1.65  0.19920
age         -0.0471  0.0286  2.71  0.09969 .
smoking     -0.5717  0.3396  2.83  0.09233 .
mppost       1.5989  1.0612  2.27  0.13190
mppre        0.6136  0.8567  0.51  0.47382
combined    -0.3930  0.2788  1.99  0.15874
para         0.3821  0.1101 12.04  0.00052 ***
---
Signif. codes:  0 '***' 0.001 '**' 0.01 '*' 0.05 '.' 0.1 ' ' 1

Estimated Scale Parameters:
           Estimate Std.err
(Intercept)     0.949   0.718

Correlation: Structure = exchangeable  Link = identity

Estimated Correlation Parameters:
     Estimate Std.err
alpha -0.00735  0.0111
Number of clusters:   429   Maximum cluster size: 19
```

Supplementary Fig. 7: Association test between CCR and parity in *MED12* tumors

```
geeglm(formula = CCR ~ age + combined + smoking + mp + para,
     family = "binomial", data = data, id = data$patient, corstr =
"exchangeable")
 Coefficients:
           Estimate  Std.err Wald Pr(>|W|)
(Intercept)  0.00882  1.30973 0.00    0.99
age         -0.00848  0.02154 0.16    0.69
combined    -0.05508  0.23177 0.06    0.81
smoking     -0.20474  0.24560 0.69    0.40
mppost       0.02078  0.47486 0.00    0.97
mppre       -0.17265  0.42792 0.16    0.69
para        -0.04146  0.08508 0.24    0.63

Estimated Scale Parameters:
           Estimate Std.err
(Intercept)     0.971  0.0391
Correlation: Structure = exchangeable  Link = identity

Estimated Correlation Parameters:
     Estimate Std.err
alpha 0.313  0.0685
Number of clusters:   303   Maximum cluster size: 14
```

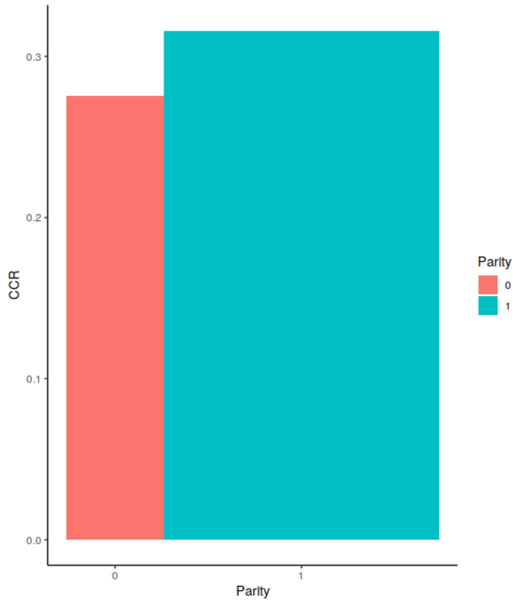

Supplementary Fig. 8: Association test between CCR and parity in WT tumors

Source data are provided as a Source Data file.

```
Call:
geeglm(formula = totalLoss ~ size + location + para, family =
poisson, data = med12, id = id.1, corstr = "exchangeable")
 Coefficients:
                     Estimate  Std.err   Wald Pr(>|W|)
(Intercept)          14.84663  0.24925 3547.91  < 2e-16 ***
size                  0.00661  0.00496   1.78   0.18276
locationSubmucous -0.45800  0.45146   1.03   0.31035
locationSubserous -1.28464  0.33868   14.39   0.00015 ***
para                  0.30306  0.09686   9.79   0.00175 **
---
Signif. codes:  0 '***' 0.001 '**' 0.01 '*' 0.05 '.' 0.1 ' ' 1

Estimated Scale Parameters:
          Estimate Std.err
(Intercept) 45740394 8042130
Correlation: Structure = exchangeable  Link = identity

Estimated Correlation Parameters:
     Estimate Std.err
alpha   0.0519  0.0199
Number of clusters:   344   Maximum cluster size: 18
```

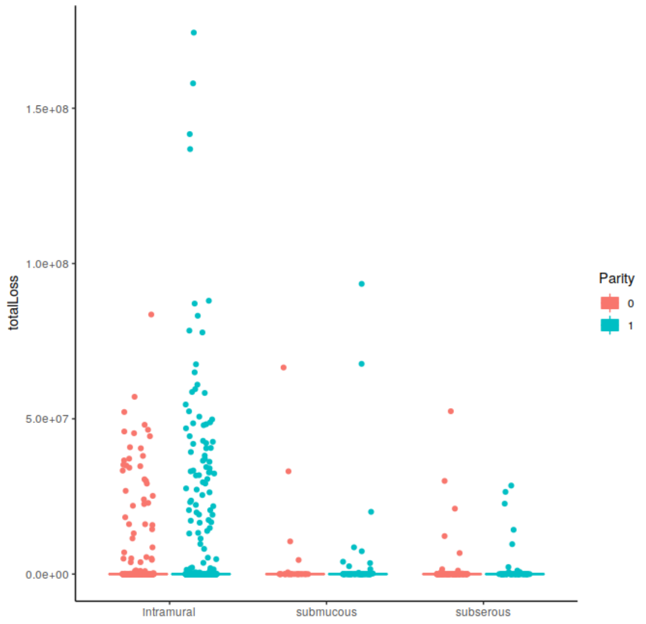

Supplementary Fig. 9: Relationship between tumor location and total loss in MED12 tumors

Intramural *MED12 n*=870 and WT *n*=269; suberous *MED12 n*=175 and WT *n*=18; submucous *MED12 n*=97 and WT *n*=22. Source data are provided as a Source Data file.

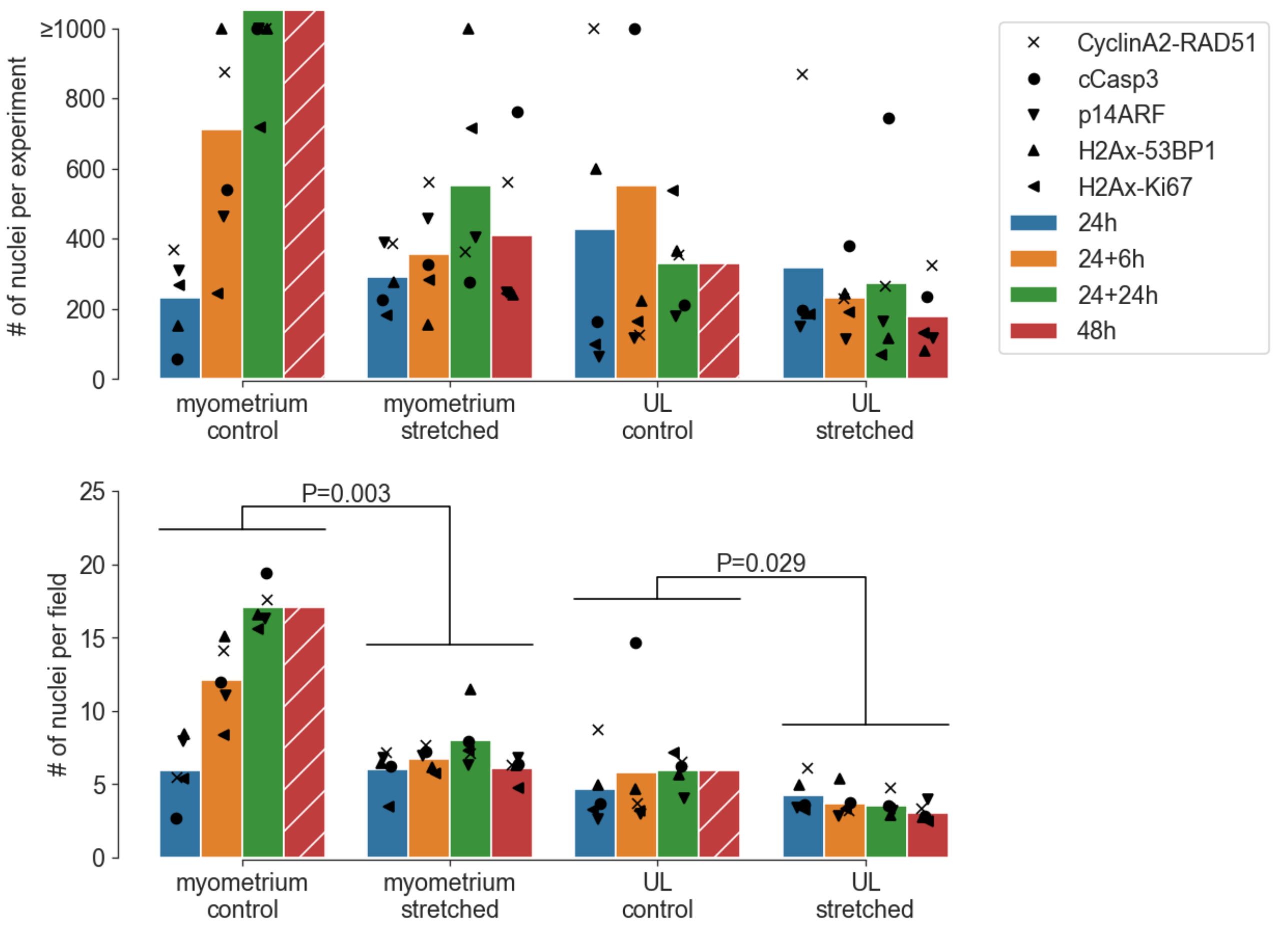

Supplementary Fig. 10: Numbers of nuclei identified in fluorescence staining image analysis

A more detailed version of Figure 4C. On top, total numbers of nuclei per experiment condition (dots) and average over the five stainings (bars). *Y*-axis values are truncated to 1,000. Stretched and non-stretched control cell cultures shown separately for both myometrium and uterine leiomyoma (UL). On bottom, average number of nuclei per 40x image field (dots) and average over the five stainings (bars). Stretched cell cultures were less dense than the non-stretched cultures in both myometrium [median numbers of nuclei per field were 12.0 and 6.6 for control (*n*=15) and stretched (*n*=20), respectively; unadjusted, two-sided Mann–Whitney U test *P*=0.003] and uterine leiomyoma [medians 4.6 and 3.3 for control (*n*=15) and stretched (*n*=20), respectively; *P*=0.029] experiments. Source data are provided as a Source Data file.

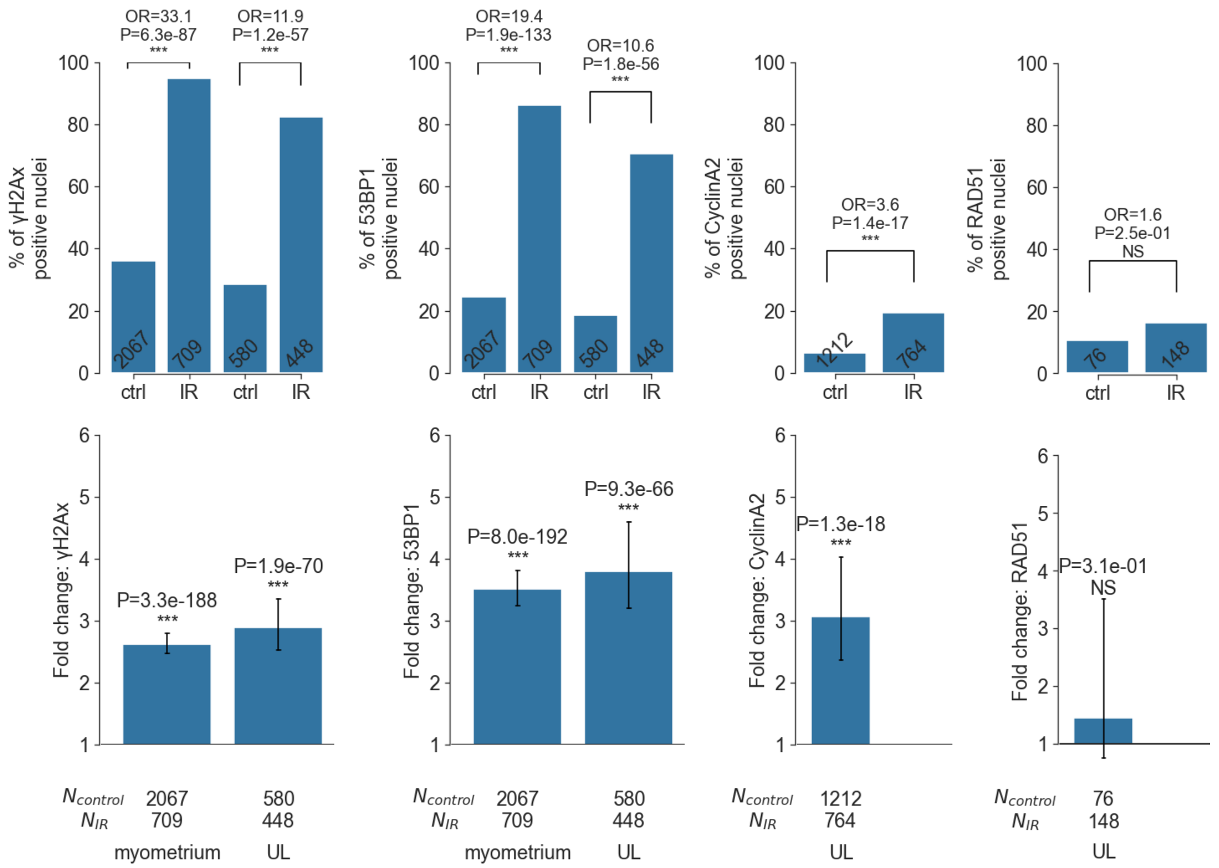

Supplementary Fig. 11: Summary of ionizing irradiation experiments

Cell markers depicted, in four columns from left to right, are γH2Ax, 53BP1 (double-positivity with γH2Ax), CyclinA2, and RAD51 (among CyclinA2-positive cells). Data are presented as a proportion (%) of positive nuclei (top bar plots) and as a fold-change of nuclei-positivity (bottom bar plots) between irradiated and control cells; error bars show a bootstrapping 95% confidence interval of each fold-change value. γH2Ax and 53BP1 display a substantial increase in DNA damage and repair upon ionizing irradiation (IR) compared to the control cells (ctrl). RAD51 was also induced upon IR, however, the numbers of cells in S/G2 (CyclinA2-positive nuclei) were not high enough to determine statistical significance. Top panels show unadjusted, two-sided *P*-values (logistic regression) and odds ratio (OR) estimates. Bottom panels shown as a reference for the resulting fold-change estimates (bar

plot) and unadjusted, two-sided *P*-values (Fisher's exact test). *** denotes $P<0.0001$; and not significant (NS) $P \geq 0.05$.

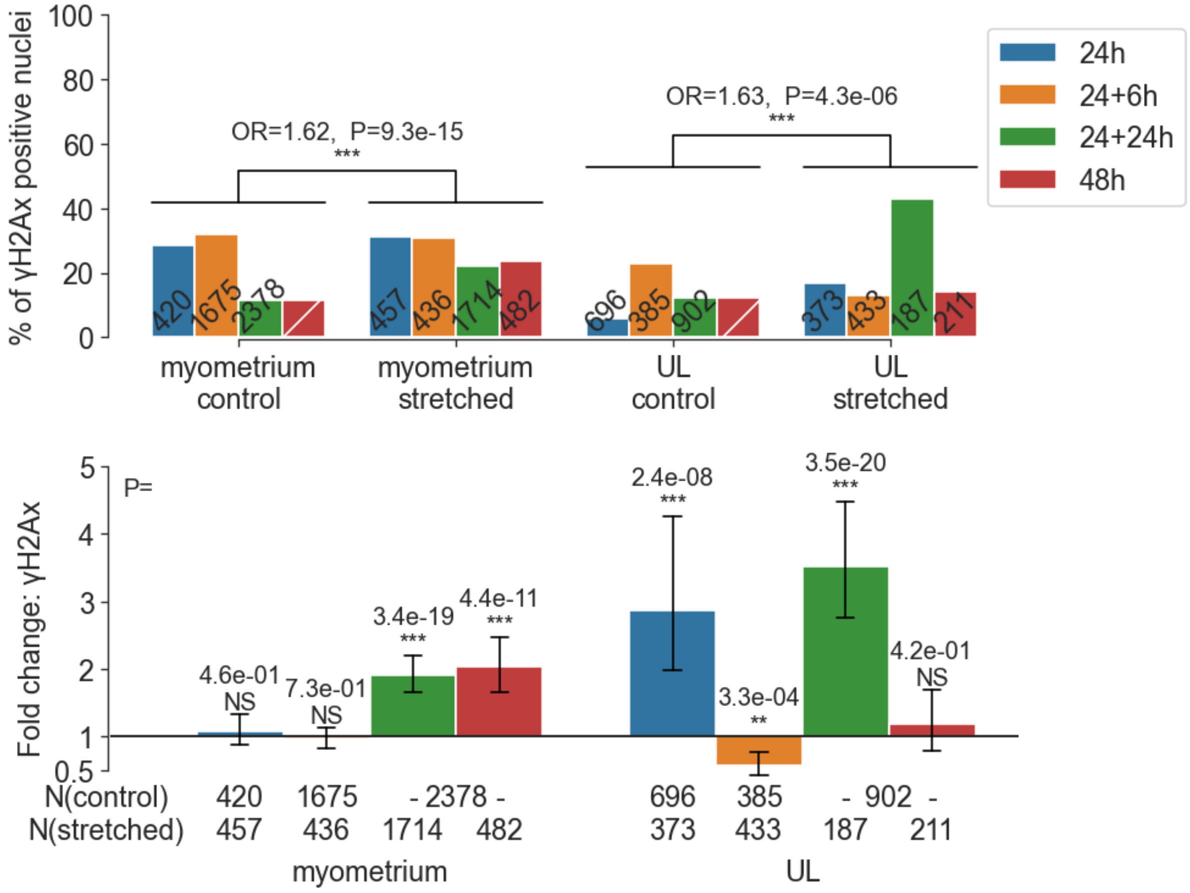

Supplementary Fig. 12: Summary of γH2Ax staining

A more detailed version of Figure 4d. Data are presented as a proportion (%) of γH2Ax-positive nuclei (top bar plots) and as a fold-change of γH2Ax-positivity (bottom bar plots) between stretched and non-stretched control cell cultures of myometrium and uterine leiomyoma (UL); error bars show a bootstrapping 95% confidence interval of each fold-change value. Top panel shows logistic regression odds-ratios (OR) and significance levels (unadjusted, two-sided *P*-values). Numbers denote total numbers of nuclei quantified in each analysis. Bottom panel shows unadjusted, two-sided *P*-values (Fisher's exact test). *** denotes $P<0.0001$; ** $P<0.001$; * $P<0.05$; and not significant (NS) $P\geq0.05$. Source data are provided as a Source Data file.

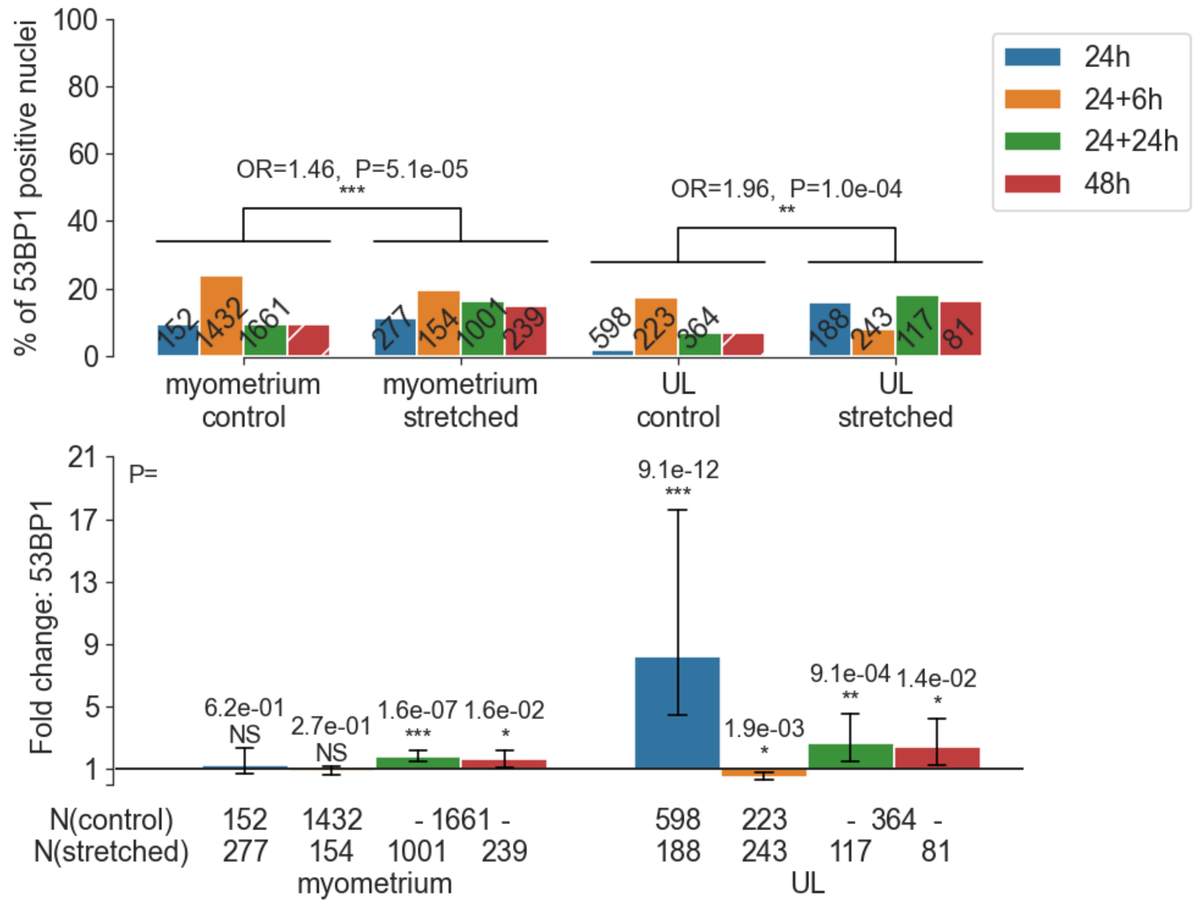

Supplementary Fig. 13: Summary of 53BP1 staining

A more detailed version of Figure 4e. Data are presented as a proportion (%) of 53BP1-positive nuclei (top bar plots) and as a fold-change of 53BP1-positivity (bottom bar plots) between stretched and non-stretched control cell cultures of myometrium and uterine leiomyoma (UL); error bars show a bootstrapping 95% confidence interval of each fold-change value. Top panel shows logistic regression odds-ratios (OR) and significance levels (unadjusted, two-sided *P*-values). Numbers denote total numbers of nuclei quantified in each analysis. Bottom panel shows unadjusted, two-sided *P*-values (Fisher's exact test). *** denotes $P<0.0001$; ** $P<0.001$; * $P<0.05$; and not significant (NS) $P\geq0.05$. Source data are provided as a Source Data file.

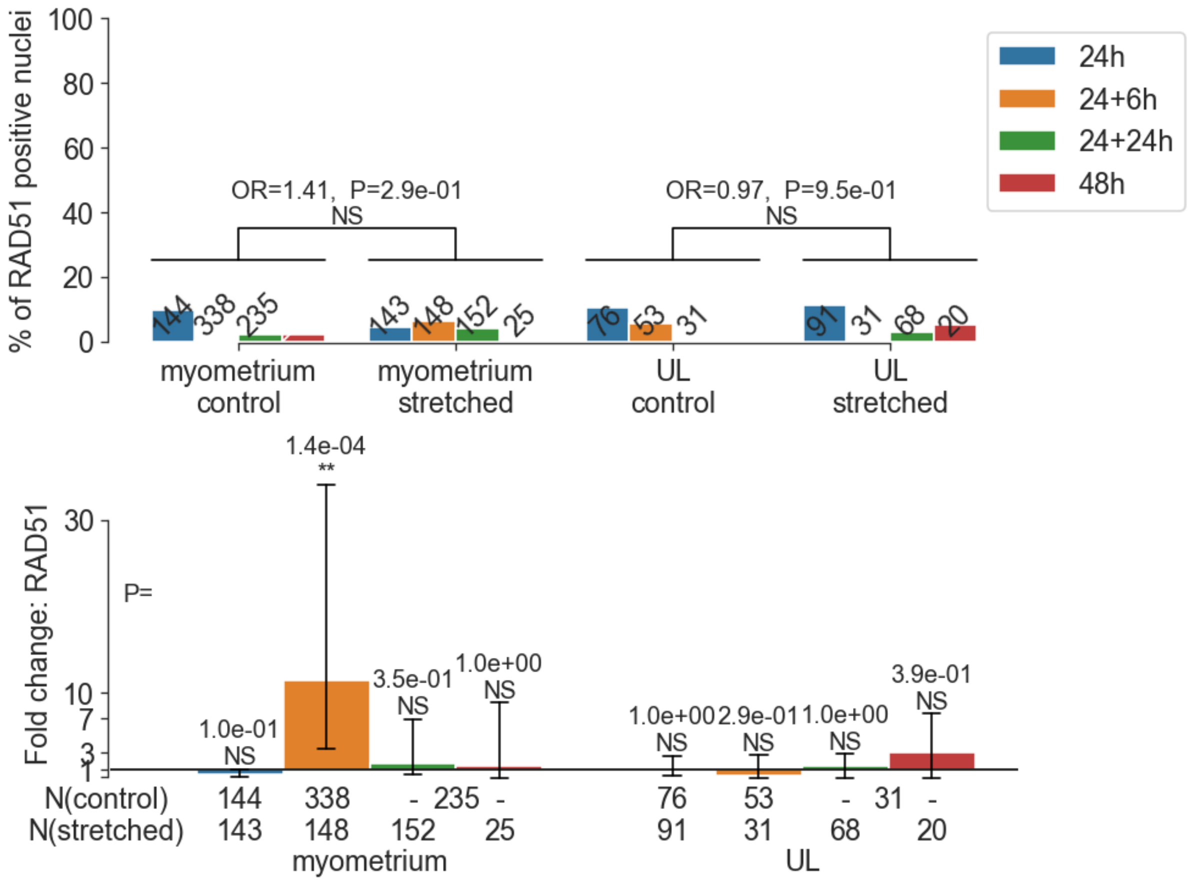

Supplementary Fig. 14: Summary of RAD51 staining

RAD51-positivity was assessed only among cells in S/G2 (CyclinA2-positive nuclei). Data are presented as a proportion (%) of RAD51-positive nuclei (top bar plots) and as a fold-change of RAD51-positivity (bottom bar plots) between stretched and non-stretched control cell cultures of myometrium and uterine leiomyoma (UL); error bars show a bootstrapping 95% confidence interval of each fold-change value. Top panel shows logistic regression odds-ratios (OR) and significance levels (unadjusted, two-sided *P*-values). Numbers denote total numbers of nuclei quantified in each analysis. Bottom panel shows unadjusted, two-sided *P*-values (Fisher's exact test). *** denotes *P*<0.0001; ** *P*<0.001; * *P*<0.05; and not significant (NS) *P*≥0.05. Source data are provided as a Source Data file.

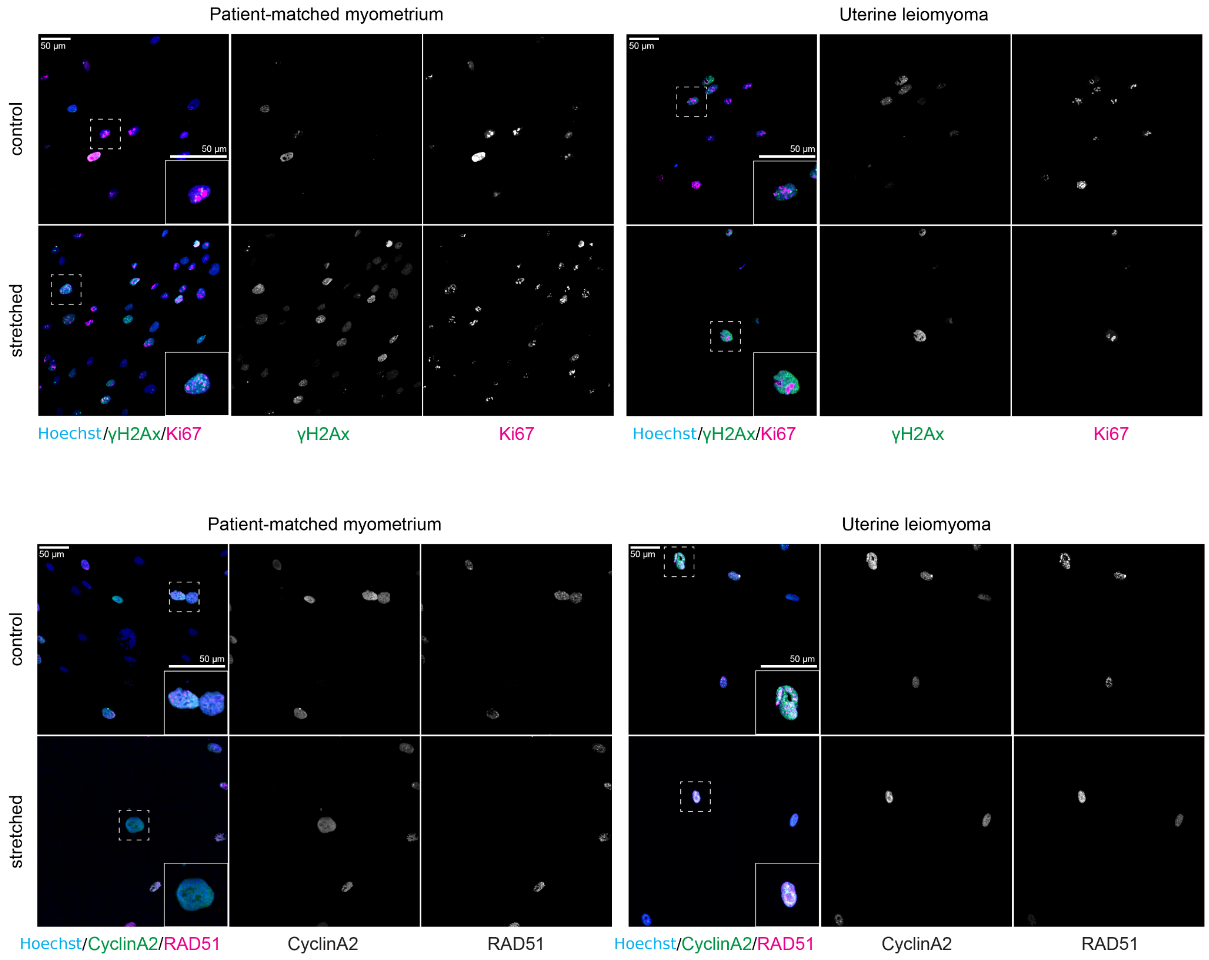

Supplementary Fig. 15: Representative images of γH2Ax, Ki67, CyclinA2 and RAD51 positivity

Representative field of view of cells under each experiment condition, stretched and non-stretched control, at the 24h+24h time point. Insets display a magnified example of an individual nucleus. Patient-matched myometrium (left column) and uterine leiomyoma (right) shown. Hoechst: nuclear staining marker; γH2Ax: DNA damage marker; Ki67: proliferation marker; CyclinA2: cell cycle marker; RAD51: DNA repair marker. The data is from a single experiment performed in parallel for primary myometrium and leiomyoma cells in duplicate culture wells per experimental condition.

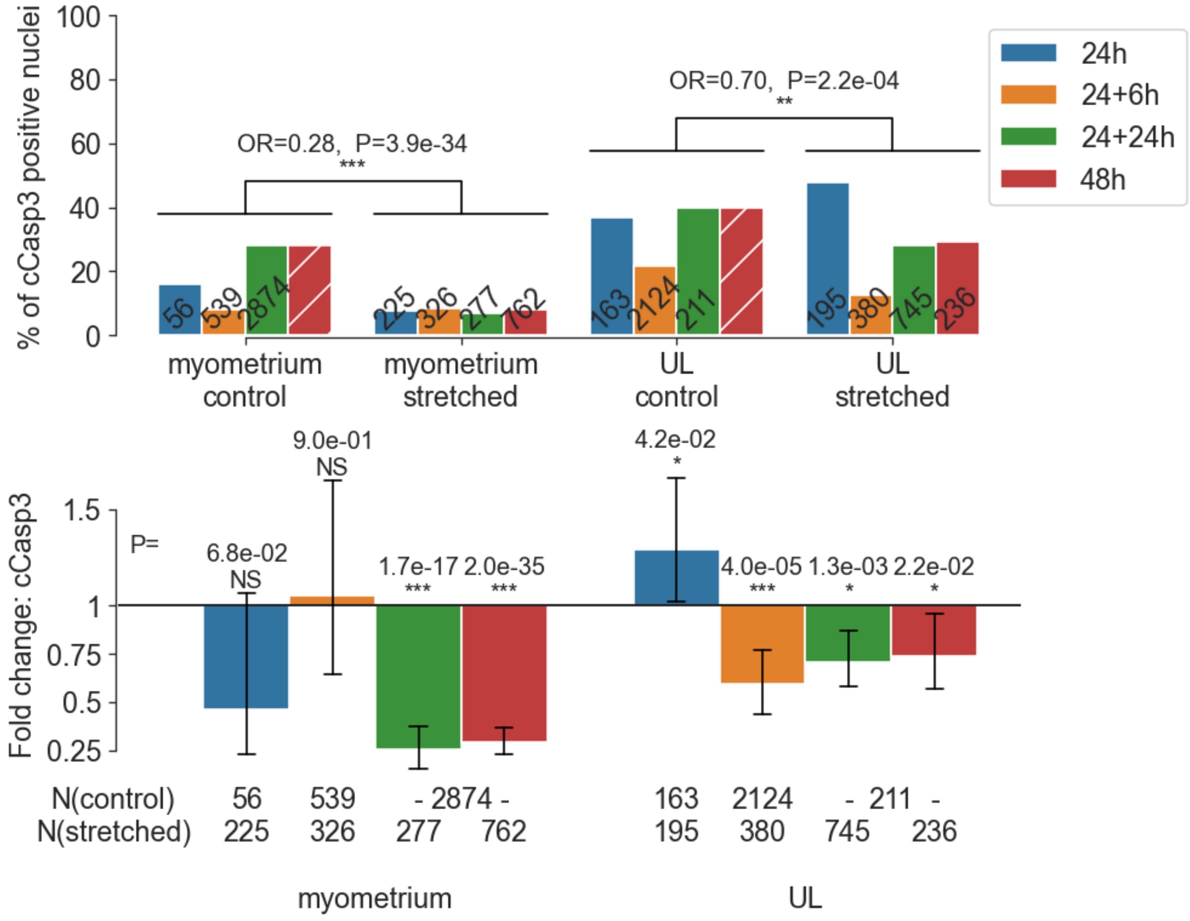

Supplementary Fig. 16: Summary of cCasp3 staining

Data are presented as a proportion (%) of cCasp3-positive nuclei (top bar plots) and as a fold-change of cCasp3-positivity (bottom bar plots) between stretched and non-stretched control cell cultures of myometrium and uterine leiomyoma (UL); error bars show a bootstrapping 95% confidence interval of each fold-change value. Top panel shows logistic regression odds-ratios (OR) and significance levels (unadjusted, two-sided *P*-values). Numbers denote total numbers of nuclei quantified in each analysis. Bottom panel shows unadjusted, two-sided *P*-values (Fisher's exact test). *** denotes $P<0.0001$; ** $P<0.001$; * $P<0.05$; and not significant (NS) $P\geq0.05$. Source data are provided as a Source Data file.

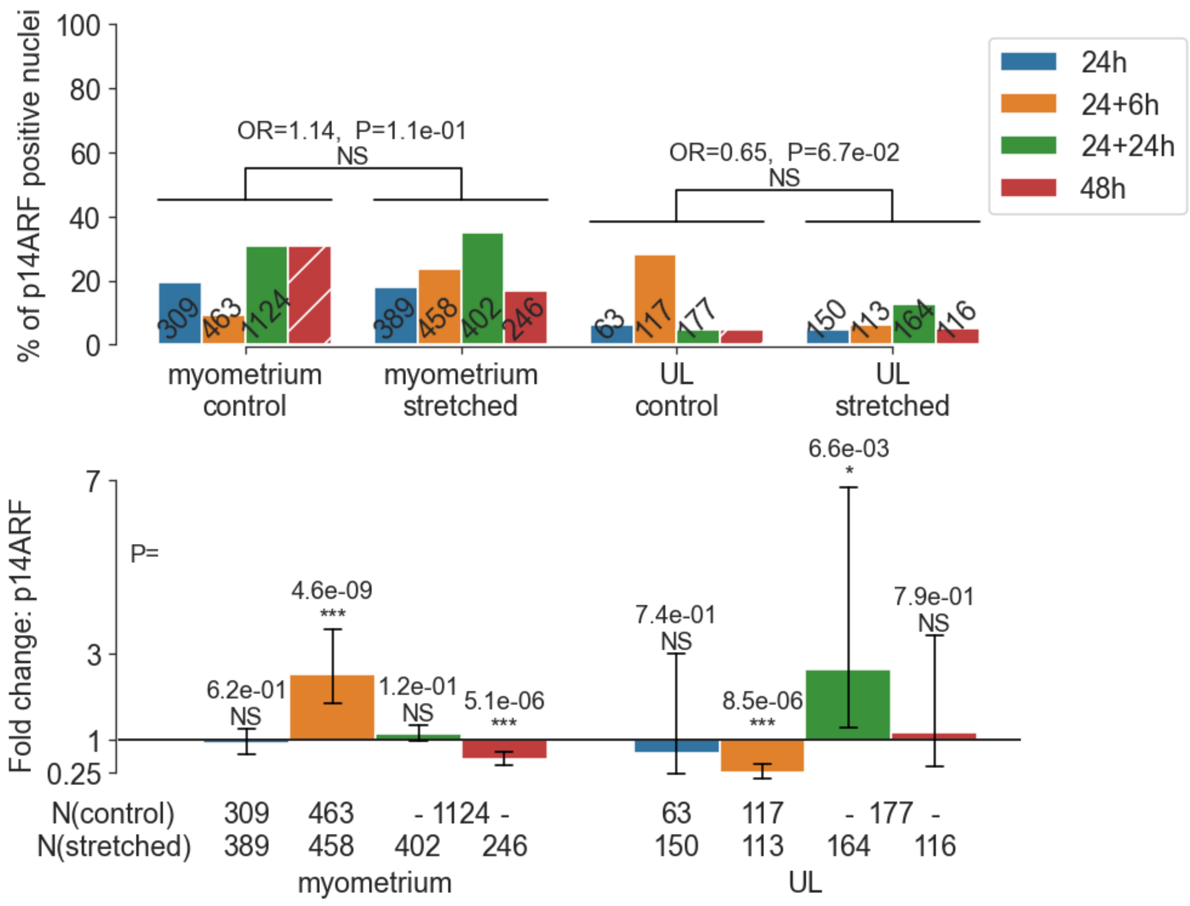

Supplementary Fig. 17: Summary of p14ARF staining

Data are presented as a proportion (%) of p14ARF-positive nuclei (top bar plots) and as a fold-change of p14ARF-positivity (bottom bar plots) between stretched and non-stretched control cell cultures of myometrium and uterine leiomyoma (UL); error bars show a bootstrapping 95% confidence interval of each fold-change value. Top panel shows logistic regression odds-ratios (OR) and significance levels (unadjusted, two-sided *P*-values). Numbers denote total numbers of nuclei quantified in each analysis. Bottom panel shows unadjusted, two-sided *P*-values (Fisher's exact test). *** denotes $P<0.0001$; ** $P<0.001$; * $P<0.05$; and not significant (NS) $P\geq0.05$. Source data are provided as a Source Data file.

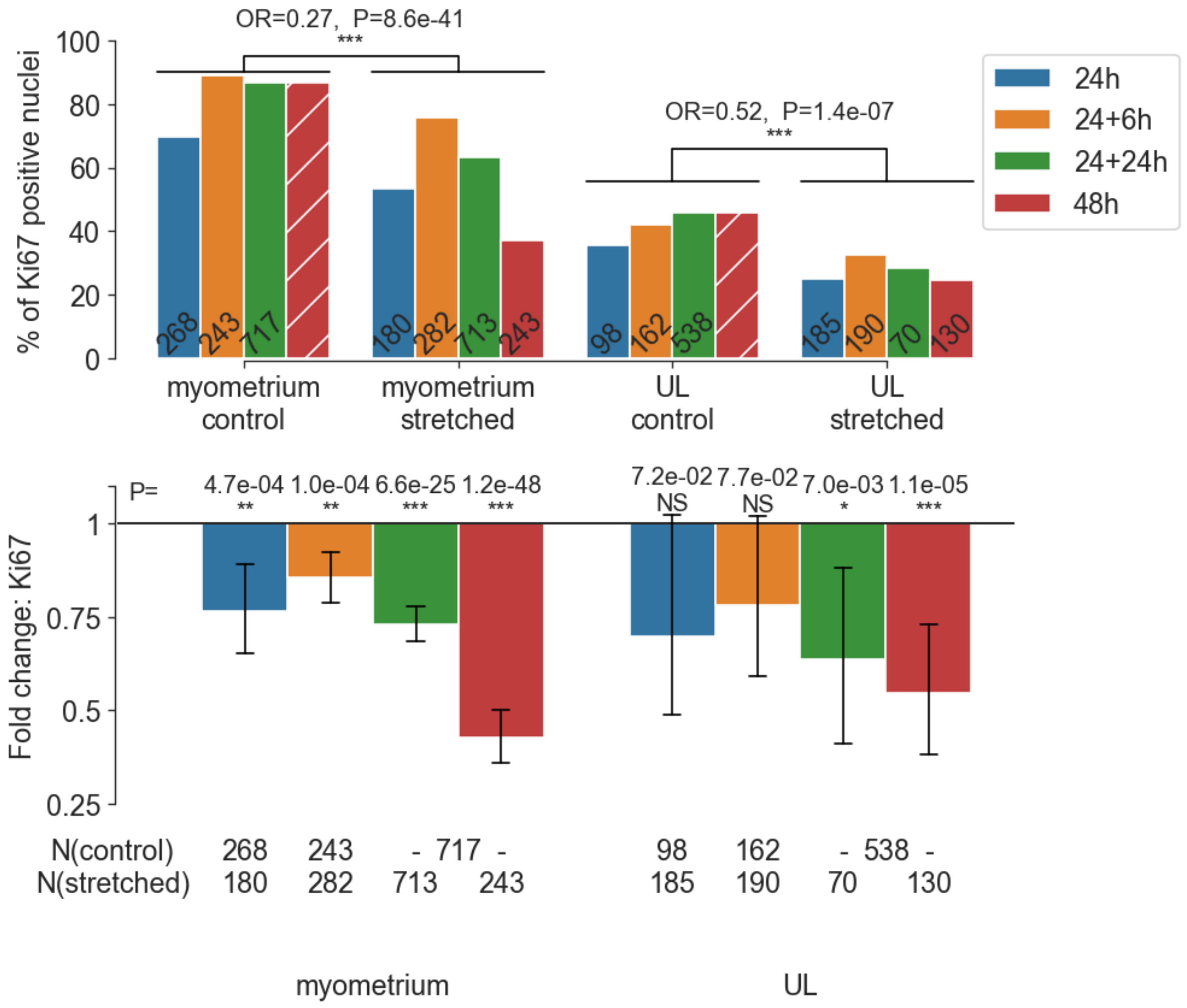

Supplementary Fig. 18: Summary of Ki67 staining

Data are presented as a proportion (%) of Ki67-positive nuclei (top bar plots) and as a fold-change of Ki67-positivity (bottom bar plots) between stretched and non-stretched control cell cultures of myometrium and uterine leiomyoma (UL); error bars show a bootstrapping 95% confidence interval of each fold-change value. Top panel shows logistic regression odds-ratios (OR) and significance levels (unadjusted, two-sided *P*-values). Numbers denote total numbers of nuclei quantified in each analysis. Bottom panel shows unadjusted, two-sided *P*-values (Fisher's exact test). *** denotes *P*<0.0001; ** *P*<0.001; * *P*<0.05; and not significant (NS) *P*≥0.05. Source data are provided as a Source Data file.

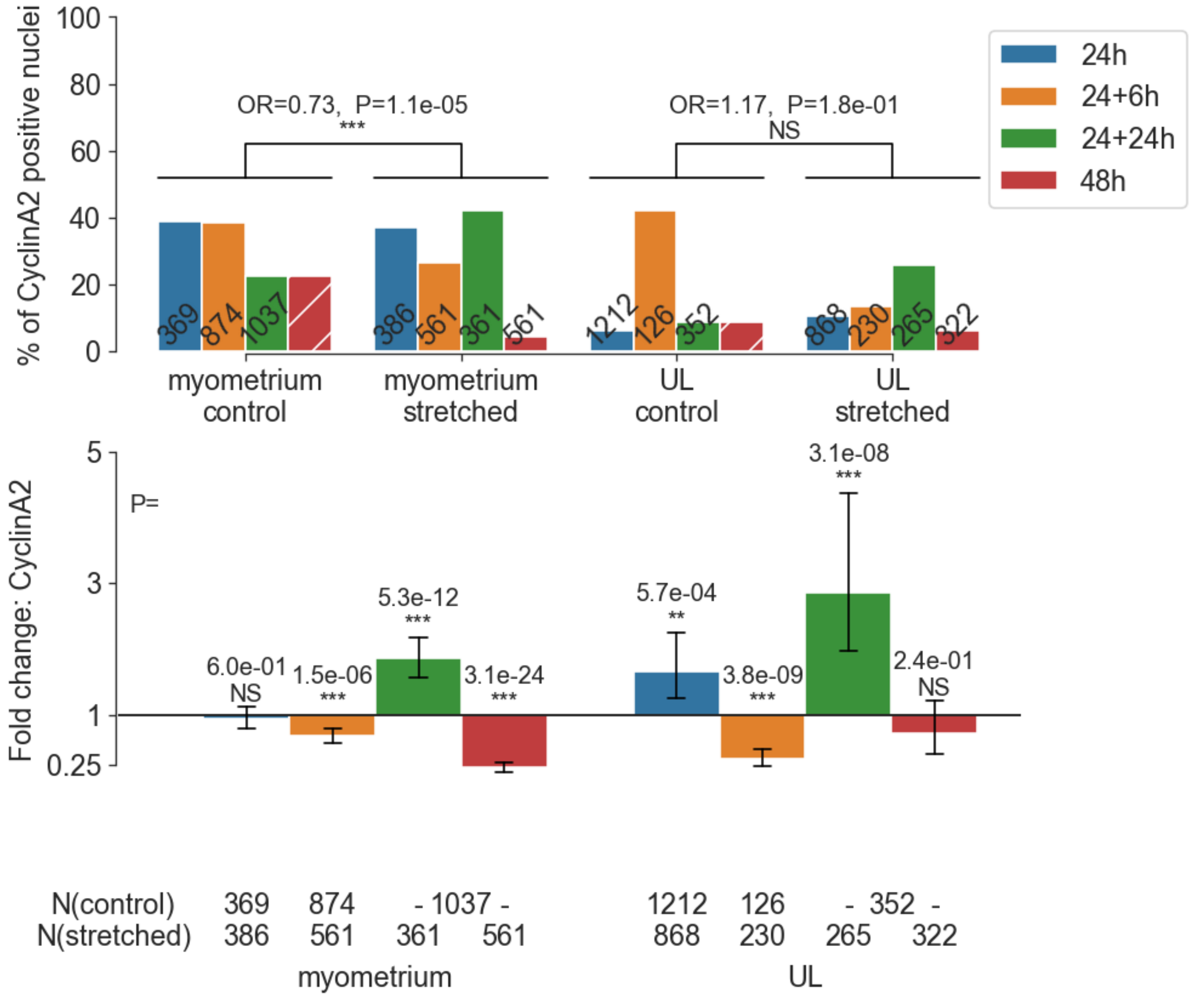

Supplementary Fig. 19: Summary of CyclinA2 staining

Data are presented as a proportion (%) of CyclinA2-positive nuclei (top bar plots) and as a fold-change of CyclinA2-positivity (bottom bar plots) between stretched and non-stretched control cell cultures of myometrium and uterine leiomyoma (UL); error bars show a bootstrapping 95% confidence interval of each fold-change value. Top panel shows logistic regression odds-ratios (OR) and significance levels (unadjusted, two-sided *P*-values). Numbers denote total numbers of nuclei quantified in each analysis. Bottom panel shows unadjusted, two-sided *P*-values (Fisher's exact test). *** denotes *P*<0.0001; ** *P*<0.001; * *P*<0.05; and not significant (NS) *P*≥0.05. Source data are provided as a Source Data file.

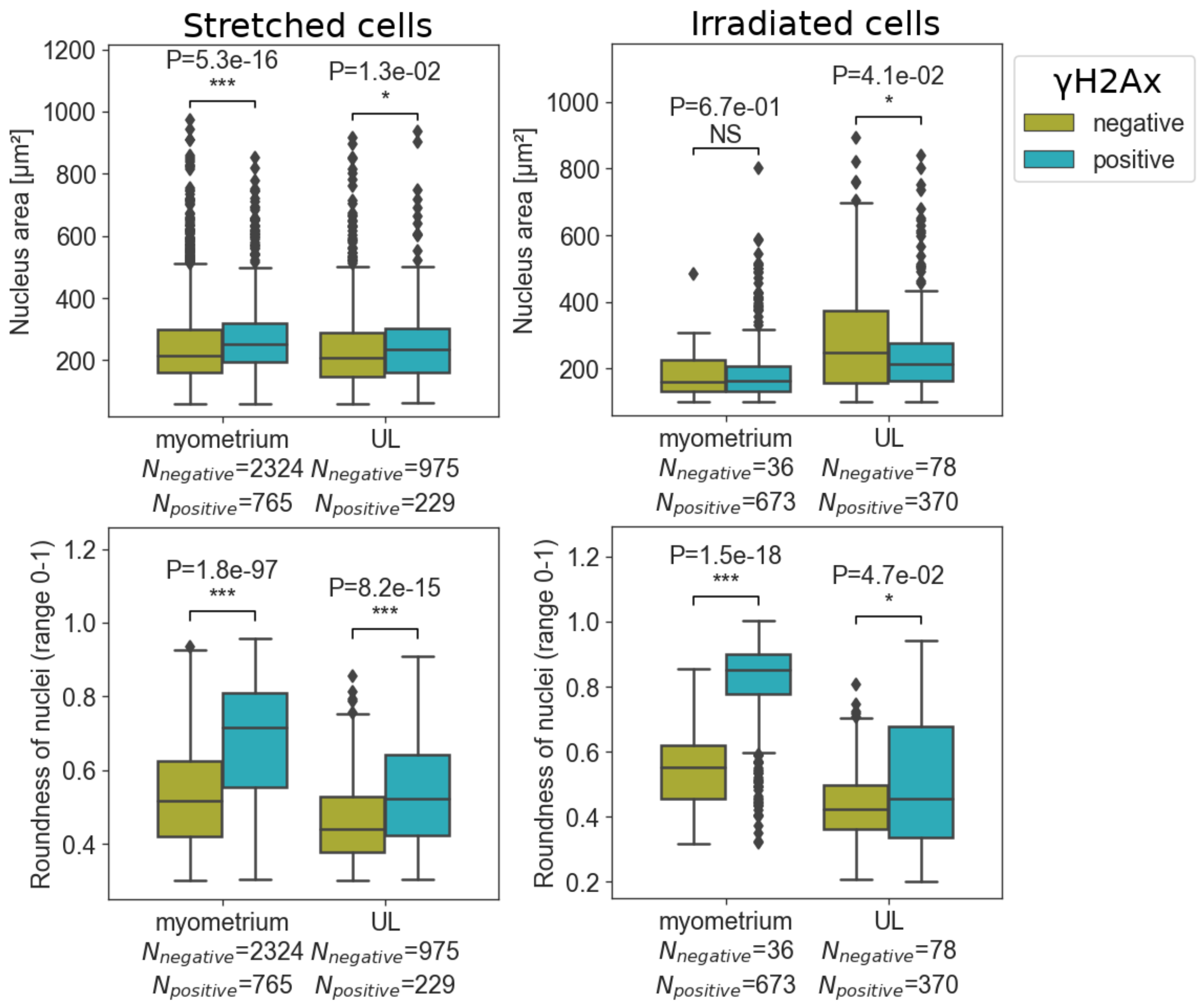

Supplementary Fig. 20: *In silico* estimates of differences in nuclear morphology

Top panels show the nucleus area measurements (µm²; *N* denotes the number of measured cells) and, bottom panels, roundness of nuclei measurements (value range 0-1, where values close to 1 denote more round nuclei). Both measurements are based on computational estimates from the *in silico* image analysis. On the left column, stretched cells and, on right, irradiated cells. UL: uterine leiomyoma. Box plots show γH2Ax-positive cells (blue) compared to γH2Ax-negative cells (green). Unadjusted, two-sided Mann–Whitney U test *P*-values shown: *** denotes $P<0.0001$; * $P<0.05$; not significant (NS) $P\geq0.05$. Box plots show the median and the first and third quartiles; error bars extend up to 1.5 IQR outside the

quartiles and any values past 1.5 IQR are drawn as dots. Source data is in Supplementary Data 4.

SUPPLEMENTARY TABLES

Supplementary Table 1: Patient characteristics

	**Parity = 0**	**Parity > 0**
N [%]	189 [32]	406 [68]
Age at hysterectomy (median) [IQR*]	47 [43-51]	49 [46-53]
Hormonal contraceptives (*N*) [%]	98 [51]	206 [50]
Smoking (*N*) [%]	64 [33]	116 [28]
Premenopausal (*N*) [%]	165 [85]	315 [73]
Hormonal replacement therapy (*N*) [%]	16 [8]	60 [14]
Postmenopausal (*N*) [%]	13 [7]	55 [13]

*interquartile range

Supplementary Table 2. Allelic imbalance characteristics of ULs (*N*=1935)

**All tumors**	***MED12* nulliparous**	***MED12* parous**	**WT nulliparous**	**WT parous**
**Number of tumors**	683	836	108	308
**CCR* %**	2.3	4.3	27.8	32
**Number of breakpoints****	0.49 (0.39-0.60)	0.66 (0.54- 0.78)	4.3 (3.10-5.58)	6.47 (5.17- 7.90)
**Total length of loss#**	3.5 (2.5-4.4)	5.6 (4.5-6.9)	43.9 (30.6-59.0)	64.6 (53.5- 77.9)
**Total length of gain#**	2.3 (1.2-3.5)	3.6 (1.8-5.9)	13.2 (4.9-26.0)	18.0 (9.7- 29.9)
**CCR tumors**	***MED12* nulliparous**	***MED12* parous**	**WT nulliparous**	**WT parous**
**Number of tumors**	16	36	29	98
**Number of breakpoints****	7.25 (5.75-9.00)	6.94 (5.94-8.17)	13 (10.93- 15.79)	18.17 (14.74-22.14)
**Total length of loss#**	28.2 (12.6-49.6)	43.8 (30.7-59.7)	100.1 (72.0- 124.9)	133.2 (108.1- 160.5)
**Total length of gain#**	12.2 (0.1-33.7)	3.9 (0.02-11.5)	13.6 (3.7-25.0)	16.8 (8.8- 24.8)
**Non-CCR tumors**	***MED12* nulliparous**	***MED12* parous**	**WT nulliparous**	**WT parous**
**Number of tumors**	667	800	79	210
**Number of breakpoints****	0.32 (0.26-0.39)	0.37 (0.31- 0.44)	1.04 (0.6-1.6)	1.07 (0.89- 1.28)
**Total length of loss#**	2.9 (2.1-3.7)	3.9 (3.1-4.9)	23.9 (11.3-39.1)	32.6 (23.8- 43.5)
**Total length of gain#**	2.0 (0.9-3.4)	3.5 (1.7-5.7)	12.9 (2.4-30.7)	18.7 (6.6- 35.8)

*CCR = complex chromosomal rearrangement

** Mean and 95% bootstrap confidence interval (CI) of the mean reported.

Mean and 95% bootstrap CI are rounded to Mbp precision

Supplementary Table 3. Confirmation of CCR by long-read whole-genome sequencing

Tumor	SNP-array breakpoints⫲	Visual inspection of WGS‡		Somatic SV counts based on WGS data†				
		CCR	Interconnected DSB	DEL	DUP	INS	INV	TR
My6290m3	112	yes	many	46	56	0	131	0
My6223m1	41	yes	1p, 12q	36	27	1	69	130
My6124m1	41	yes	many	17	13	0	27	43
My6107m1	40	yes	10q, 13q	15	12	6	38	71
My8018m1	37	yes	many	26	12	0	36	67
My6322m1	32	yes	many	19	13	2	18	23
My6310m1	32	yes	many	21	14	0	39	26
My6306m1	32	yes	6q, 14q	9	4	0	11	44
My6037m1	26	yes	2p	31	19	5	57	11
My6250m1	25	no		4	2	0	1	2
My6211m2	24	yes	1p, 6q	9	5	0	10	2
My6056m1*	22	yes	11q	15	18	13	18	13
My6022m1	22	yes	2q, 4q	7	3	1	12	9
My6110m1	20	yes	5p, 11q	20	9	28	24	13
My6091m1	20	yes	6p, 14q	3	1	6	4	18
My6011m1	20	yes	22q	5	4	0	6	10
My6180m14	18	yes	21q	0	1	0	3	16
My5019m1	18	yes	8p	1	3	0	4	9
My6271m1	17	yes	3q	6	1	0	3	3
My6038m1*	16	yes	8p, 14q	9	2	8	6	13
My6063m1	16	yes	8q, 8p	14	5	4	18	7
My6198m5	14	yes	2p, 15q	30	2	6	7	10
My1010m1	14	yes	5q	7	4	0	14	13
My1008m2	14	no		3	0	0	0	1
My6191m1	13	yes	5q, Xq, 19q	0	1	0	2	10
My8016m1#	12	yes	11p	10	7	3	16	2
My6168m3	12	yes	14q, 19p	31	30	1	50	26
My6161m8	12	yes	6q	1	1	0	4	1
My6033m1	12	yes	Xp, Xq	17	15	7	38	0
My6286m1	11	yes	17q, 22q	7	0	0	4	8
My6191m3	11	yes	1p	0	1	0	0	2
My6013m3*	10	yes	12p	23	3	8	9	11
My8016m2#	10	yes	11p, 11q	11	7	2	9	0
My6447m1	10	yes	4q	5	2	0	7	2
My6334m2	10	yes	5q, 7q	4	0	2	6	13
My6125m1	10	no		6	0	0	1	3
My6125m2	9	yes	5q	7	3	1	3	1
My6244m2	8	yes	7q	7	0	1	6	1
My6338m1	6	yes	2q	6	0	2	5	0
My6303m1	6	yes	1q	5	1	0	5	0
My6267m1	6	yes	6p, Xp, 4p, 14q	3	0	0	3	2
My6127m7	6	yes	7q	1	0	1	3	0
My6118m1	4	yes	12q, 14q	4	1	0	0	2

ǂ Estimated number of breakpoints based on the SNP-array data.

‡ Visual inspection of the WGS data was carried out systematically over all 43 tumors to identify any somatic complex chromosomal rearrangements (CCR; “yes”: at least one CCR was found; “no”: no evidence of CCRs). The chained double-strand breaks (DSB) were found on these chromosome arms; see the circos plots in Supplementary Fig. 5 for a genome-wide overview.

† Numbers of somatic structural variants (DEL: deletions; DUP: duplications; INS: insertions; INV: inversions; TR: inter-chromosomal translocations).

* The corresponding normal tissue sample did not pass sequencing quality for three tumors.

# One clonally related pair of tumors was included in WGS, My8016m1 and My8016m2.

Supplementary Table 4. The effect of tumor location to allelic imbalance

**Response (bp)**	**Tumor location***	**Tumors**	***N* tumors**	***N* patients**	***P* # (adjusted)**	**Model**	**Effect [95% CI] (adjusted)**
Total AI region length	submucous	*MED12*	1,142	344	1	Poisson	0.54 [0.16 - 1.7]
Total AI region length	subserous	*MED12*	1,142	344	1	Poisson	0.6 [0.22-1.6]
Total loss AI region length	submucous	*MED12*	1,142	344	1	Poisson	0.63 [0.18-2.28]
Total loss AI region length	subserous	*MED12*	1,142	344	0.00165	Poisson	0.28 [0.11-0.72]

*In addition to tumor location, parity and tumor size were used as confounders. Intramural location is the reference level. AI: allelic imbalance.

#Bonferroni adjusted two-sided, poisson regression *P*-values and 95% confidence intervals (adjusted alpha 0.05/11).

Supplementary Table 5. Numbers of nuclei identified in fluorescence staining image analysis

Cell culture	Time point	Condition †	CyclinA2, RAD51	cCasp3	p14ARF	γH2Ax, 53BP1	γH2Ax, Ki67
Uterine leiomyoma	24h	ctrl	1212	163	63	598	98
		stretch	868	195	150	188	185
	24+6h	ctrl	126	2124	117	223	162
		stretch	230	380	113	243	190
	24+24h	ctrl	352	211	177	364	538
		stretch	265	745	164	117	70
	48h *	stretch	322	236	116	81	130
	IR ‡	ctrl	-	-	-	580	-
		irradiated	764	-	-	448	-
Myometrium	24h	ctrl	369	56	309	152	268
		stretch	386	225	389	277	180
	24+6h	ctrl	874	539	463	1432	243
		stretch	561	326	458	154	282
	24+24h	ctrl	1037	2874	1124	1661	717
		stretch	361	277	402	1001	713
	48h *	stretch	561	762	246	239	243
	IR ‡	ctrl	-	-	-	2067	-
		irradiated	-	-	-	709	-

* The control for the 48h time point was the same as in the 24+24h time point.

‡ Ionizing irradiation (IR) experiments were stained only for a subset of markers.

† Experiment condition was either stretched (“stretch”), irradiated (IR experiments only) or non-stretched control (“ctrl” for short).

Supplementary Table 6. DNA damage and repair in stretched and non-stretched cells

Marker	Cells *	Control (non-stretched) cells *		Stretched Cells *		Fisher's exact test †		Logistic regression ǂ (adjusted for covariates: time point and, for γH2Ax, the secondary marker)				
		Negative	Positive	Negative	Positive	OR	*P*	OR	OR 95% CI		*P* ‡	*Q* ‡
γH2Ax	myometrium	3548	925	2324	765	1.3	3.2E-05	1.62	1.44	1.84	9.3E-15	**5.6E-14**
	UL	1746	237	975	229	1.7	6.8E-08	1.63	1.32	2.00	4.3E-06	**2.2E-05**
53BP1	myometrium	2733	512	1411	260	1.0	8.7E-01	1.46	1.22	1.75	5.1E-05	**2.0E-04**
	UL	1110	75	546	83	2.2	1.8E-06	1.96	1.39	2.75	1.0E-04	**3.1E-04**
RAD51	myometrium	697	20	447	21	1.6	1.4E-01	1.41	0.75	2.66	2.9E-01	5.8E-01
	UL	149	11	197	13	0.9	8.3E-01	0.97	0.41	2.30	9.5E-01	9.5E-01

* UL: uterine leiomyoma. Numbers of positive and negative cells identified in the *in silico* image analysis of control and stretched cells.

† Two-sided Fisher's exact test (*P*) and the resulting odds-ratio (OR) are shown as a reference.

ǂ Logistic regression test of marker positivity between stretched and control cells, adjusted for covariates: the three time-points (24h, 30h and 48h) and, for γH2Ax, the secondary marker (see Methods for details on the experiment design). Odds-ratio (OR) and its 95% confidence interval (OR 95% CI, low and high values). OR values >1 suggest that stretching increases marker positivity.

‡ Two-sided significance level of the logistic regression test (*P*; unadjusted) and the resulting multiple-testing adjusted *P*-values (*Q*; Holm's method). Adjusted *P*-values that passed *Q*<0.05 are shown bolded.

Supplementary Table 7. DNA damage and repair in each time point

Marker	Cells †	Time point †	Control (non-stretched) cells *			Stretched cells *			FC ǂ	FC 95% CI ǂ		*P* ‡	*Q* ‡
			Negative	Positive	%	Negative	Positive	%					
γH2Ax	myomet.	24h	300	120	29%	315	142	31%	1.09	0.89	1.34	4.6E-01	1.0E+00
		24+6h	1143	532	32%	302	134	31%	0.97	0.82	1.13	7.3E-01	1.0E+00
		24+24h	2105	273	11%	1338	376	22%	1.91	1.66	2.20	3.4E-19	**7.7E-18**
		48h				369	113	23%	2.05	1.67	2.47	4.4E-11	**9.2E-10**
	UL	24h	656	40	6%	311	62	17%	2.86	1.97	4.24	2.4E-08	**4.8E-07**
		24+6h	297	88	23%	376	57	13%	0.58	0.43	0.78	3.3E-04	**5.7E-03**
		24+24h	793	109	12%	107	80	43%	3.52	2.77	4.49	3.5E-20	**8.4E-19**
		48h				181	30	14%	1.20	0.79	1.71	4.2E-01	1.0E+00
53BP1	myomet.	24h	138	14	9%	246	31	11%	1.18	0.68	2.33	6.2E-01	1.0E+00
		24+6h	1091	341	24%	124	30	19%	0.83	0.57	1.13	2.7E-01	1.0E+00
		24+24h	1504	157	9%	837	164	16%	1.73	1.41	2.12	1.6E-07	**3.0E-06**
		48h				204	35	15%	1.57	1.09	2.16	1.6E-02	2.1E-01
	UL	24h	587	11	2%	158	30	16%	8.16	4.51	17.9	9.1E-12	**2.0E-10**
		24+6h	184	39	17%	224	19	8%	0.46	0.26	0.76	1.9E-03	**2.8E-02**
		24+24h	339	25	7%	96	21	18%	2.60	1.50	4.53	9.1E-04	**1.5E-02**
		48h				68	13	16%	2.37	1.19	4.41	1.4E-02	1.9E-01
RAD51	myomet.	24h	130	14	10%	137	6	4%	0.47	0.14	1.09	1.0E-01	1.0E+00
		24+6h	337	1	1%	139	9	6%	11.33	3.40	34.0	1.4E-04	**2.5E-03**
		24+24h	230	5	2%	146	6	4%	1.80	0.51	6.93	3.5E-01	1.0E+00
		48h				25	0	1%	1.46	0.00	8.78	1.0E+00	1.0E+00
	UL	24h	68	8	11%	81	10	11%	1.03	0.42	2.68	1.0E+00	1.0E+00
		24+6h	50	3	6%	31	0	1%	0.42	0.00	2.78	2.9E-01	1.0E+00
		24+24h	31	0	1%	66	2	3%	1.41	0.00	2.83	1.0E+00	1.0E+00
		48h				19	1	5%	3.00	0.00	7.50	3.9E-01	1.0E+00

† UL: uterine leiomyoma; myomet.: myometrium; time points 24+24h and 48h share the same control experiment.

* Numbers of positive and negative cells identified in the *in silico* image analysis of control and stretched cells, and the resulting proportions (%) of marker-positive cells.

ǂ Fold-change (FC) of marker-positivity and its 95% confidence interval (FC 95% CI, low and high values; bootstrapping with 10000 resamples). FC values >1 suggest that stretching increases marker positivity.

‡ Two-sided Fisher's exact test (*P*; unadjusted) and the resulting multiple-testing adjusted *P*-values (*Q*; Holm's method). Adjusted *P*-values that passed *Q*<0.05 are shown bolded.

Supplementary Table 8. Summary of other markers in stretched and non-stretched cells

Marker	Cells †	Time point †	Control (non-stretched) cells *			Stretched cells *			FC ⱡ	FC 95% CI ⱡ		*P* ‡
			Negative	Positive	%	Negative	Positive	%				
CyclinA2	myomet.	24h	225	144	39%	243	143	37%	0.95	0.79	1.13	6.0E-01
		24+6h	536	338	39%	413	148	26%	0.68	0.58	0.80	1.5E-06
		24+24h	802	235	23%	209	152	42%	1.86	1.57	2.18	5.3E-12
		48h				536	25	4%	0.20	0.13	0.29	3.1E-24
	UL	24h	1136	76	6%	777	91	10%	1.67	1.24	2.25	5.7E-04
		24+6h	73	53	42%	199	31	13%	0.33	0.22	0.47	3.8E-09
		24+24h	321	31	9%	197	68	26%	2.86	1.97	4.44	3.1E-08
		48h				302	20	6%	0.72	0.40	1.22	2.4E-01
Ki67	myomet.	24h	81	187	70%	84	96	53%	0.77	0.65	0.89	4.7E-04
		24+6h	27	216	89%	68	214	76%	0.85	0.79	0.93	1.0E-04
		24+24h	95	622	87%	261	452	63%	0.73	0.69	0.78	6.6E-25
		48h				153	90	37%	0.43	0.36	0.50	1.2E-48
	UL	24h	63	35	36%	139	46	25%	0.70	0.49	1.02	7.2E-02
		24+6h	94	68	42%	128	62	33%	0.78	0.59	1.02	7.7E-02
		24+24h	292	246	46%	50	20	29%	0.64	0.42	0.89	7.0E-03
		48h				98	32	25%	0.55	0.39	0.73	1.1E-05
cCasp3	myomet.	24h	47	9	16%	208	17	8%	0.46	0.22	1.05	6.8E-02
		24+6h	496	43	8%	299	27	8%	1.05	0.65	1.65	9.0E-01
		24+24h	2069	805	28%	258	19	7%	0.26	0.15	0.37	1.7E-17
		48h				700	62	8%	0.29	0.23	0.37	2.0E-35
	UL	24h	103	60	37%	102	93	48%	1.29	1.02	1.68	4.2E-02
		24+6h	1667	457	22%	332	48	13%	0.60	0.43	0.77	4.0E-05
		24+24h	127	84	40%	536	209	28%	0.70	0.58	0.87	1.3E-03
		48h				167	69	29%	0.74	0.56	0.95	2.2E-02
p14ARF	myomet.	24h	249	60	19%	320	69	18%	0.91	0.67	1.26	6.2E-01
		24+6h	420	43	9%	350	108	24%	2.50	1.84	3.54	4.6E-09
		24+24h	778	346	31%	261	141	35%	1.14	0.97	1.33	1.2E-01
		48h				205	41	17%	0.55	0.40	0.72	5.1E-06
	UL	24h	59	4	6%	143	7	5%	0.68	0.21	2.78	7.4E-01
		24+6h	84	33	28%	106	7	6%	0.24	0.09	0.46	8.5E-06
		24+24h	169	8	5%	143	21	13%	2.64	1.31	6.47	6.6E-03
		48h				110	6	5%	1.18	0.38	3.34	7.9E-01

† UL: uterine leiomyoma; myomet.: myometrium; time points 24+24h and 48h share the same control experiment.

* Numbers of positive and negative cells identified in the *in silico* image analysis of control and stretched cells, and the resulting proportions (%) of marker-positive cells.

ⱡ Fold-change (FC) of marker-positivity and its 95% confidence interval (FC 95% CI, low and high values; bootstrapping with 10000 resamples). FC values >1 suggest that stretching increases marker positivity.

‡ Two-sided Fisher's exact test *P*-values (*P*; unadjusted).

Supplementary Table 9. Comparison of complex chromosomal rearrangement (CCR) status between WGS and SNP-array

**sample**	**WGS**	**SNP-array**	**Note**
B7m6	NO	NA	
M12m1	**NO**	**NO**	
M17m1	**YES**	**YES**	
M18m1	YES	NO	Interconnected events in WGS
M1m3	**NO**	**NO**	
M29m2	**YES**	**YES**	
M32m1	**NO**	**NO**	
M32m8	**NO**	**NO**	
M38m5	**YES**	**YES**	
M44m1	**YES**	**YES**	
M44m2	**YES**	**YES**	
M49m1	**NO**	**NO**	
M4m3	**NO**	**NO**	
M5m1	**NO**	**NO**	
M68m1	**NO**	**NO**	
My10m3	YES	NA	
My16m1	NO	NA	
My18m1	**YES**	**YES**	
My18m2	**YES**	**YES**	
My18m3	**YES**	**YES**	
My1m1	NO	YES	The deletions are not interconnected. This is detectable only using WGS-data
My22m1	YES	NO	Low number of interconnected events in WGS
My23m1	**NO**	**NO**	
My23m2	**NO**	**NO**	
My23m3	**YES**	**NO**	One of the SNP-array regions is slightly too short
My23m4	YES	NA	
My24m3	**NO**	**NO**	
My29m1	**NO**	**NO**	
My30m1	YES	NO	Low number of interconnected events in WGS
My33m2	**NO**	**NO**	
My45m5	**NO**	**NO**	
My46m1	**YES**	**YES**	
My47m1	**YES**	**YES**	
My48m1	**NO**	**NO**	
My64m1	**YES**	**YES**	
My64m2	**NO**	**NO**	
My9m3	**NO**	**NO**	
N7m1	NO	NA	

Supplementary Table 10. List of antibodies

	Supplier/catalog number	Dilution
Mouse anti-ɣH2Ax	Abcam ab22551	1:1000
Rabbit anti-53BP1	Abcam ab36823	1:500
Mouse anti-CyclinA2	GeneTex GT2547	1:1000
Rabbit anti-RAD51	Abcam ab133534	1:1000
Rabbit anti-Ki67	Abcam ab15580	1:1000
Rabbit anti-cleaved Caspase3	Cell Signaling Technologies #9664	1:300
Rabbit anti-p14ARF	Abcam ab216602	1:500
Alexa Fluor 488-Phalloidin	Molecular Probes A12379	1:40
