# Supplementary material for: Parity associates with chromosomal damage in uterine leiomyomas
Source: Nat Commun. 2021 Sep 14;12:5448. doi: 10.1038/s41467-021-25806-x (PMC8440576; doi:10.1038/s41467-021-25806-x)
Supplement: Supplementary file 2 — Description of Additional Supplementary Files [file 41467_2021_25806_MOESM2_ESM.pdf]

Description of Additional Supplementary Files

File Name: Supplementary Data 1
Description: Somatic allelic imbalance segment coordinates, Log R Ratio (LRR), and mirrored B-allele frequency (mBAF) for each tumor.

File Name: Supplementary Data 2
Description: Patient specific variables (menopause status, smoking status, age, and use of oral contraceptives) and tumor-specific variables (subtype, location, CCR status, breakpoint number, and allelic imbalance length).

File Name: Supplementary Data 3
Description: Harmony software parameters utilized in the imaging analysis.

File Name: Supplementary Data 4
Description: Quantified imaging data.
