# Supplementary material for: Parity associates with chromosomal damage in uterine leiomyomas
Source: Nat Commun. 2021 Sep 14;12:5448. doi: 10.1038/s41467-021-25806-x (PMC8440576; doi:10.1038/s41467-021-25806-x)
Supplement: Supplementary file 7 — Reporting Summary [file 41467_2021_25806_MOESM7_ESM.pdf]

natureresearch

Corresponding author(s): Lauri Aaltonen

Last updated by author(s): Aug 17, 2021

Reporting Summary

Nature Research wishes to improve the reproducibility of the work that we publish. This form provides structure for consistency and transparency in reporting. For further information on Nature Research policies, see Authors & Referees and the Editorial Policy Checklist.

Statistics

For all statistical analyses, confirm that the following items are present in the figure legend, table legend, main text, or Methods section.

n/a	Confirmed	
☐	☒	The exact sample size (*n*) for each experimental group/condition, given as a discrete number and unit of measurement
☐	☒	A statement on whether measurements were taken from distinct samples or whether the same sample was measured repeatedly
☐	☒	The statistical test(s) used AND whether they are one- or two-sided<br>*Only common tests should be described solely by name; describe more complex techniques in the Methods section.*
☐	☒	A description of all covariates tested
☐	☒	A description of any assumptions or corrections, such as tests of normality and adjustment for multiple comparisons
☐	☒	A full description of the statistical parameters including central tendency (e.g. means) or other basic estimates (e.g. regression coefficient) AND variation (e.g. standard deviation) or associated estimates of uncertainty (e.g. confidence intervals)
☐	☒	For null hypothesis testing, the test statistic (e.g. *F*, *t*, *r*) with confidence intervals, effect sizes, degrees of freedom and *P* value noted<br>*Give P values as exact values whenever suitable.*
☒	☐	For Bayesian analysis, information on the choice of priors and Markov chain Monte Carlo settings
☐	☒	For hierarchical and complex designs, identification of the appropriate level for tests and full reporting of outcomes
☐	☒	Estimates of effect sizes (e.g. Cohen's *d*, Pearson's *r*), indicating how they were calculated

Our web collection on statistics for biologists contains articles on many of the points above.

Software and code

Policy information about availability of computer code

Data collection

No software was used for the data collection.

Data analysis

SNP array analysis:
All tumor and normal pairs were genotyped with Infinium HumanCore-24 (Illumina) SNP arrays. B-allele frequencies (BAF) and log-R ratios (LRR) were extracted with Illumina GenomeStudio software (version 2011.1; Genotyping module v1.9). After GC wave adjustment of the data with PennCNV (version 1.0.4). allelic imbalance regions were calculated for all tumors using BAF segmentation (v1.2.0).

Long read WGS sequencing:
Sequencing and basecalling was performed on PromethION platform using MinKnow-Live-Basecalling (version 3.4.6). The basecalled reads were aligned with minimap2 (v2.16; preset: map-ont) against the GRCh38 reference genome (GCA_000001405.15, excluding alt contigs). Data quality was inspected with NanoStat (v1.1.2) and NanoPlot (v1.20.0). Each aligned long-read library was then processed with Sniffles (v1.0.11; min_support: 2, min_length: 10, num_reads_report: 2, min_seq_size: 1000) to identify an initial set of structural variants (SV). The initial SV calls from all tumors and all normals were merged together with SURVIVOR (v1.0.6; max. 50bp distance between breakpoints). Systematic, manual inspection was carried out with BasePlayer (October 2019 version).

Imaging data was analysed with Harmony software (v4.9 , PerkinElmer)

Statistical analysis was performed using R with packages geepack (v. 1.2-1), boot (v. 1.3-20), epiR (v. 1.0-4), survival (v. 2.38) and DHARMa (v. 0.4.1).

See full details in the Methods section.

For manuscripts utilizing custom algorithms or software that are central to the research but not yet described in published literature, software must be made available to editors/reviewers. We strongly encourage code deposition in a community repository (e.g. GitHub). See the Nature Research guidelines for submitting code & software for further information.

Data

Policy information about availability of data

All manuscripts must include a data availability statement. This statement should provide the following information, where applicable:

- Accession codes, unique identifiers, or web links for publicly available datasets
- A list of figures that have associated raw data
- A description of any restrictions on data availability

The somatic allele imbalance segment data generated in this study are provided in Supplementary Data 1. The tumor- and patient-specific variables used in this study are provided in Supplementary Data 2. The Harmony software parameters used in this study are provided in Supplementary Data 3. The quantified imaging data generated in this study are provided in Supplementary Data 4. The GRCh38 reference genome data used in this study are available in the GenBank database under accession code GCA_000001405.15 [https://www.ncbi.nlm.nih.gov/assembly/GCF_000001405.26/]. Source data are provided with this paper.

Field-specific reporting

Please select the one below that is the best fit for your research. If you are not sure, read the appropriate sections before making your selection.

☒ Life sciences ☐ Behavioural & social sciences ☐ Ecological, evolutionary & environmental sciences

For a reference copy of the document with all sections, see nature.com/documents/nr-reporting-summary-flat.pdf

Life sciences study design

All studies must disclose on these points even when the disclosure is negative.

Sample size	No statistical methods were used to predetermine the sample size. We aimed at a large sample size, in thousands rather than hundreds of ULs, to ensure appropriate representation of different types of samples. Prospectively collected hysterectomy samples from six sample collections were utilized in the study; altogether 2,131 uterine leiomyomas (ULs) and 690 corresponding normal myometrium tissue samples. For the registry analysis, all suitable Finnish uterine leiomyosarcoma cases were used. The sample size was sufficient to reach the aims and make the presented conclusions.
Data exclusions	11 tumors that had common clonal origin were excluded from the SNP array analysis using criteria established during the study. In addition, patients lacking background information (parity, age, smoking, oral contraceptive use, menopause status) were not used in the association test. In the registry analysis, 8 patients were excluded from the analysis due to birth place being abroad or unknown, preventing matching, and 75 were excluded due to lack of any suitable controls. Due to previous linking of nulliparity as a major risk factor in breast, ovarian and uterine cancers, the analysis set was further restricted to parous patients with at least one parous control, leaving 399 cases and 1657 controls.
Replication	The concept of mechanical stretching induced DNA damage was replicated successfully with an in vitro experiment. The quantified imaging data is shared to ensure future reproducibility of the findings. The experimental data from mechanical stretching are from a single multi time point experiment performed in parallel for primary myometrium and myoma cells in duplicate culture wells per experimental condition.
Randomization	Relevant background variables (age, menopause status, smoking status, use of oral contraceptives) were used as confounders in the statistical analysis. The cultured myoma and myometrium cells were derived from the same individual for all experimental conditions.
Blinding	Blinding was not relevant to the parts of the study that did not utilize experimental groups. For the experiments in cultured cells, quantification of fluorescent stainings was performed using an unbiased automated image analysis in Harmony 4.9 software.

Reporting for specific materials, systems and methods

We require information from authors about some types of materials, experimental systems and methods used in many studies. Here, indicate whether each material, system or method listed is relevant to your study. If you are not sure if a list item applies to your research, read the appropriate section before selecting a response.

Materials & experimental systems

n/a	Involved in the study	
☐	☒	Antibodies
☒	☐	Eukaryotic cell lines
☒	☐	Palaeontology
☒	☐	Animals and other organisms
☐	☒	Human research participants
☒	☐	Clinical data

Methods

n/a	Involved in the study	
☒	☐	ChIP-seq
☒	☐	Flow cytometry
☒	☐	MRI-based neuroimaging

Antibodies

Antibodies used	Mouse anti-ɣH2Ax Abcam ab22551 (dilution: 1:1000)<br>Rabbit anti-53BP1 Abcam ab36823 (dilution: 1:500)<br>Mouse anti-CyclinA2 GeneTex GT2547 (dilution: 1:1000)<br>Rabbit anti-RAD51 Abcam ab133534 (dilution: 1:1000)<br>Rabbit anti-Ki67 Abcam ab15580 (dilution: 1:1000)<br>Rabbit anti-cleaved Caspase3 Cell Signaling Technologies #9664 (dilution: 1:300<br>Rabbit anti-p14ARF Abcam ab216602 (dilution: 1:500)<br>Alexa Fluor 488-Phalloidin Molecular Probes A12379 (dilution: 1:40)<br>goat anti-mouse Alexa 488 secondary antibody, Invitrogen A11029 (dilution 1:1000)<br>goat anti-rabbit Alexa 647 secondary antibody, Invitrogen A21245 (dilution 1:1000)
Validation	Abcam ab22551: Species reactivity: Human; manufacturer's Abpromise guarantee covers the use of ab22551 in the following tested applications: includes immunocytochemistry.<br><br>Abcam ab36823: Species reactivity: Mouse, Human; Tested applications: WB, IHC-P, IP.<br><br>GeneTex GT2547: Species reactivity: Human; Tested applications: include immunocytochemistry/immunofluorescence.<br><br>Abcam ab133534: Species reactivity: Mouse, Rat, Human; manufacturer's Abpromise guarantee covers the use of ab133534 in the following tested applications: includes immunocytochemistry/immunofluorescence.<br><br>Abcam ab15580: Species reactivity: Mouse, Human; manufacturer's Abpromise guarantee covers the use of ab15580 in the following tested applications: includes immunocytochemistry.<br><br>Cell Signaling Technologies #9664: Species reactivity: Human, Mouse, Rat, Monkey; Tested applications: include Immunofluorescence (Immunocytochemistry); validation statement "Cleaved Caspase-3 (Asp175) (5A1) Rabbit mAb detects endogenous levels of the large fragment (17/19 kDa) of activated caspase-3 resulting from cleavage adjacent to Asp175. This antibody does not recognize full length caspase-3 or other cleaved caspases."<br><br>Abcam ab216602: Species reactivity: Human; manufacturer's Abpromise guarantee covers the use of ab216602 in the following tested applications: includes immunocytochemistry/immunofluorescence.<br><br>Molecular Probes A12379: Not an antibody; a high-affinity filamentous actin (F-actin) probe (a bicyclic peptide belonging to a family of toxins isolated from the deadly Amanita phalloides). Tested applications: can be used to visualize and quantitate F-actin in tissue sections, cell cultures, or cell-free preparations.

Human research participants

Policy information about studies involving human research participants

Population characteristics	All the participants were female who underwent hysterectomy and had at least one uterine leiomyoma. The median age of a patient was 49. 32% of the participants were nulliparous. 51% used oral hormonal contraceptives. 30% had ever smoked. 77% were premenopausal, 12% used hormonal replacement therapy and 11% were postmenopausal.<br><br>In the registry analysis cases diagnosed with ICD-O-3 topography code 54.2 or 54.9, and morphology code 8890/3 and their birth place and birth date matched healthy controls were used.
Recruitment	The sample set consists of six prospectively collected sample series (M, My, My1000, My5000, My6000 and My8000). The anonymous M-sample series was collected according to Finnish laws and regulations after authorization from the director of the health care unit, between the years 2001 and 2002. For all subsequent samples, a written informed consent was obtained. Participants were not compensated. See full details of sample collection in the Methods section. Self-selection bias did not affect the study. For the registry analysis, all suitable uterine leiomyosarcoma cases were used (see above sections for sample exclusion criteria)
Ethics oversight	The study was conducted in accordance with the Declaration of Helsinki and approved by the Finnish National Supervisory Authority for Welfare and Health, National Institute for Health and Welfare (THL/151/5.05.00/2017, THL/723/5.05.00/2018), and the Ethics Committee of the Hospital District of Helsinki and Uusimaa (HUS/2509/2016).

Note that full information on the approval of the study protocol must also be provided in the manuscript.
